# Ciliary Dyneins and Dynein Related Ciliopathies

**DOI:** 10.3390/cells10081885

**Published:** 2021-07-25

**Authors:** Dinu Antony, Han G. Brunner, Miriam Schmidts

**Affiliations:** 1 Center for Pediatrics and Adolescent Medicine, University Hospital Freiburg, Freiburg University Faculty of Medicine, Mathildenstrasse 1, 79106 Freiburg, Germany; dinu.antony@uniklinik-freiburg.de; 2 Genome Research Division, Human Genetics Department, Radboud University Medical Center, Geert Grooteplein Zuid 10, 6525 KL Nijmegen, The Netherlands; han.brunner@radboudumc.nl; 3 Radboud Institute for Molecular Life Sciences (RIMLS), Geert Grooteplein Zuid 10, 6525 KL Nijmegen, The Netherlands

**Keywords:** cilium, dynein, intraflagellar transport, primary ciliary dyskinesia, short rib polydactyly syndrome

## Abstract

Although ubiquitously present, the relevance of cilia for vertebrate development and health has long been underrated. However, the aberration or dysfunction of ciliary structures or components results in a large heterogeneous group of disorders in mammals, termed ciliopathies. The majority of human ciliopathy cases are caused by malfunction of the ciliary dynein motor activity, powering retrograde intraflagellar transport (enabled by the cytoplasmic dynein-2 complex) or axonemal movement (axonemal dynein complexes). Despite a partially shared evolutionary developmental path and shared ciliary localization, the cytoplasmic dynein-2 and axonemal dynein functions are markedly different: while cytoplasmic dynein-2 complex dysfunction results in an ultra-rare syndromal skeleto-renal phenotype with a high lethality, axonemal dynein dysfunction is associated with a motile cilia dysfunction disorder, primary ciliary dyskinesia (PCD) or Kartagener syndrome, causing recurrent airway infection, degenerative lung disease, laterality defects, and infertility. In this review, we provide an overview of ciliary dynein complex compositions, their functions, clinical disease hallmarks of ciliary dynein disorders, presumed underlying pathomechanisms, and novel developments in the field.

## 1. Enigmatic Cilia

In 1674, Antoni van Leeuwenhoek, while observing protozoans under his primitive microscope, saw some intriguing moving structures which he called “thin feet or little legs”. These enigmatic cilia, discovered centuries, ago still continue to fascinate scientists [1].

Cilia are microtubule-based organelles projecting from almost all cell types. Cilia are classified into two main groups based on their function—motile and non-motile cilia. Motile cilia, as the name suggest, help in fluid movement, locomotion of an organism, or the movement of gametes during fertilization. Non-motile cilia or primary cilia in turn functions as the main signaling hub of cells. Cilia vary in length (1 µm to 60 µm) [2,3] depending on the cell type, and have a diameter of approximately 0.25 µm [4]; however, for each cell type, the cilia length is tightly regulated. Cilia are structurally highly similar to sperm flagella; however sperm reach a far longer length [5].

The origin of cilia centers around the following three main hypotheses: (1) The symbiosis relationship theory by Lynn Sagan speculated that amoeboflagellates emerged via the symbiosis between spirochaetes and ancestral amoeboides, serving as precursor for the complex eukaryotic flagellum [6]. (2) Cavalier Smith opposed this claim by arguing that eukaryotic flagella are structurally different from prokaryotic flagella. He proposed that the loss of the cell wall, a major characteristic of eukaryotes, acted as a selection pressure, resulting in the development of cytoskeletal structures with actin and tubulin, suggesting that the ancient flagellum might have formed via the perpendicular localization of a microtubule nucleation center in relation to the cell membrane [7]. (3) Satir proposed that cilia might have originated from a cart wheel shaped enveloped RNA virus invading the bacterial cytoplasm [8]. Irrespective of the arguments about the origin of eukaryotic cilia, the ciliary structure is evolutionarily highly conserved from simple organisms like *Chlamydomonas reinhardtii* (unicellular algae), *Paramecium, Tetrahymena, Trypanosoma,* and *Caenorhabditis elegans,* to vertebrates like zebrafish, mouse, and humans [9].

Cilia formation in vertebrates is closely linked to cell cycle exit. The mother centriole is transformed into a basal body acting as an anchoring unit, allowing axonemal extension [10]. Both motile and non-motile cilia are made up of a microtubule cytoskeleton arranged in an organized fashion. While basal bodies contain nine triplet microtubules, the ciliary axoneme is made up of nine doublet microtubules. The doublet axonemal microtubules consist of a complete “A” microtubule with 13 protofilaments, and a partial “B” microtubule with 10 protofilaments [11]. In motile cilia, the doublet microtubules are arranged in 9 + 2 fashion, with nine peripheral and two central microtubules. In non-motile cilia and the embryonic nodal, motile cilia, the central microtubules are absent [12]. The protofilaments are made of stable tubulin hetero-dimers [13]. Tubulin further undergoes various post-translational modifications like acetylation, arginylation, detyrosination, glycosylation, glutamylation, ubiquitylation, palmitoylation, phosphorylation, and methylation [14], all of which are important for maintaining the structure and function of cilia [15].

Cilia do not have a protein synthesis machinery; hence, ciliary proteins need to be transported to the ciliary base and are constantly shuttled in and out of the cilia. This applies to structural ciliary components as well as various signaling molecules. The bidirectional movement along the ciliary axoneme is termed as intraflagellar transport (IFT) [16]. The plus-end directed kinesin-2 motor enables the transport of proteins to the tip of the cilia (anterograde transport), and dynein-2 motors or IFT dynein enables the transport of proteins back to the ciliary base from tip (retrograde transport) [17,18]. Attached to the motors are large multi-protein complexes serving as cargo adaptor bases, the so-called IFT complexes. IFT-B is a complex made up of 16 subunits (IFT20, 22, 25, 27, 38, 46, 52, 54, 57, 56, 70, 74, 80, 81, 88, and 172) and the IFT-A complex has 6 subunits (IFT43, 121, 122, 139, 140, and 144) [19]. The BBSome, another large multi-protein complex, functions as a selective adaptor between the IFT complex and the cargo to be transported during IFT [20,21].

**Figure 1:**
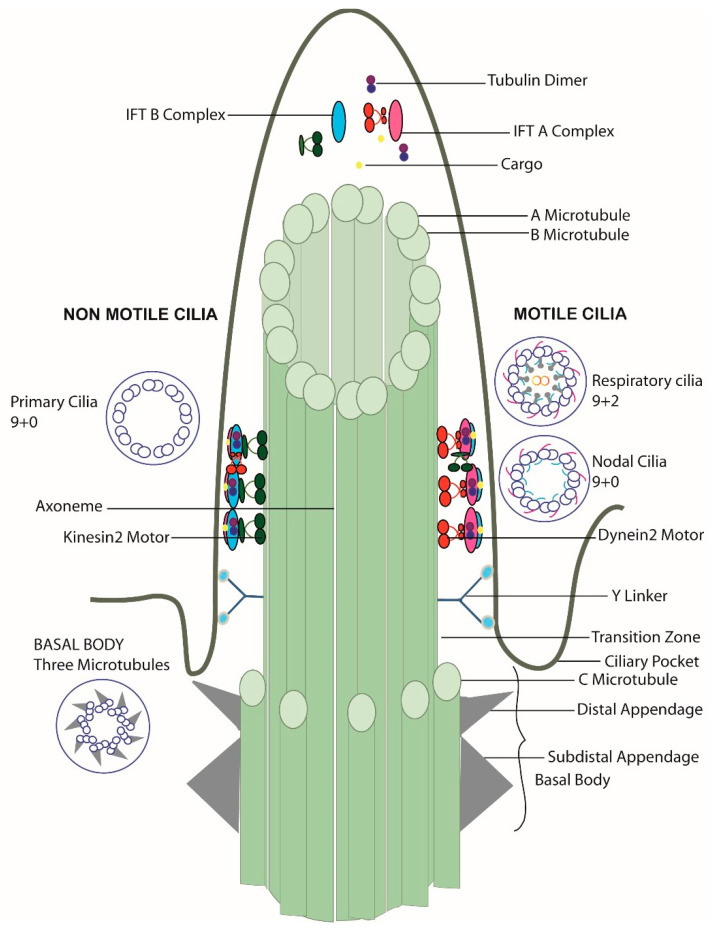
Structure of the cilia. Primary cilia with a 9 + 0 microtubule arrangement lacking dynein arms are usually immotile; respiratory epithelium cilia with a 9 + 2 microtubule arrangement with dynein arms, radial spokes, and a nexin dynein regulatory structure generate organized wave form movement while nodal cilia with a 9 + 0 microtubule arrangement with dynein arms generate a propelling rotational movement. Basal bodies show a triplet microtubule pattern.

The entrance and exit of the protein complexes in the cilia are censored at the transition zone, which acts like a gatekeeper at the border between the cilium and rest of the cell. In addition to a size filter (proteins above 50 kD cannot pass through the transition zone), the transition zone also acts as a qualitative filter. The peripheral microtubules here are connected to the ciliary membrane by Y shaped structures called Y-links. The ciliary membrane around this region is termed the ciliary necklace. The distal appendages of the basal body transform into transition zone appendages [22]. Near the base of the cilia, the plasma membrane forms an invagination, which is termed the ciliary pocket [23]. The basic structure of the cilium along with the variations seen in different types of cilia are summarized in Figure 1.

## 2. Primary Cilia

### 2.1. Primary (Non-Motile) Cilia Function

The term “primary cilia” originated from Sorokin in 1968 when he observed a solitary appendage or rudimentary cilium emerging on the surface of epithelial cells of mammalian lungs before the onset of multiciliogenesis [24]. Primary cilia were longtime considered as solitary structures with no function, as they lacked movement. However, an association between primary cilia and polycystic kidney (PKD) disease was established by Pazour et al. in 2000 when investigating a mouse deficient for the IFT component IFT88. The mouse mutant presented with kidney cysts and the cilia in those kidneys were found to be shorter than the cilia in wildtype kidneys [25,26]. This finding opened up a novel primary cilia research field. Hence, the apprehension regarding the role of the once forgotten vestigial organelle started to change.

Primary cilia are dynamic organelles, constantly adapting to intracellular and extracellular signals [27]. They can be found on the surface of nearly all mammalian cells with the exception of blood cells. Primary cilia lining the kidney tubules, epithelial cells on biliary duct and ductal cells of pancreas can bend in response to fluid flow, which stimulates various signaling pathways like calcium and purinergic signaling pathways essential for the optimal physiological function of organs. Dysfunction of primary cilia results in multiple developmental defects including cyst formations, fibrosis, skeletal abnormalities, craniofacial defects [4] neural tube defects, cerebellar vermis hypoplasia and photoreceptor defects [28,29]. The role of primary cilia as a cellular signaling hub is emphasized by their ability to regulate Hedgehog (Hh), Wnt, Platelet-derived growth factor (PDGF), Notch, G-Protein coupled receptor (GPCR), Mammalian target of rapamycin (mTOR), Transforming growth factor beta (TGF-β) and Calcium [2,30] signaling pathways.

Cilia adopt specialized structures when it comes to perception of sensory signals [31]. Vertebrate photoreceptor cells possess a specialized primary cilium restructured as the so-called outer segment consisting of tightly packed membrane discs. In addition, the outer segment is connected to the inner segment by a so-called connecting cilium, thought to represent a modified transition zone. The constant movement of signaling molecules through the connecting cilium as well as transport of “debris” back to the cell body is essential for the proper function of photoreceptor cells [31]. Primary cilia in olfactory neuronal cilia adorned with GPCR signal receptors enable perception of smell by enhancing the expression of adenylate cyclase III in neurons via GPCR signaling [31]. The single kinocilium located on mammalian hair cells is important for establishing the polarity of stereocilia or sterovilli bundles and hence contributing towards the hearing process in mammals [32]. In the developing mammalian embryo, primary cilia of the peripheral cells of the embryonic node are thought to respond to the flow generated by motile cilia and activate calcium signaling essential for nodal pathway regulation, which is crucial for vertebrate left right body axis determination [33]. There are two main perceptions by which initial body asymmetry is initiated in the node. The embryonic node contains two main types of cells, the peripherally located non-motile cilia bearing crown cells and pit cells, with motile cilia which are located in the center. According to the first model, the coordinated circular movement of the cilia of the pit cells creates a morphogen gradient towards the left side. This in turn triggers differential gene expression, hence determining organ asymmetry [34]. Most of the laterality disorder research is centered on the second two cilia model. According to this hypothesis, the movement of the motile cilia of the pit cells activates receptors and ion channels of non-motile cilia including PKD2, resulting in a calcium ion flux only on left side of the node, which in turn results in asymmetric gene expression [33].

### 2.2. Primary Cilia Structure

Primary cilia length varies from 1–10 µm for most cells except for olfactory cells where cilia can reach 60 µm [2,3]. Primary cilia lack the motility structures like dynein arms, nexin dynein regulatory complex and radial spokes (Figure 1). Cryo-electron tomography analysis of primary cilia by Sun et al. suggests that the 9 + 0 arrangement only occurs from basal body to region where the axoneme reaches the cell surface. From there, two peripheral microtubules can translocate to center of the cilium and result in a 7 + 2 arrangement, more rarely also an 8 + 1 arrangement. A fibrous protein network connecting microtubules between themselves as well as microtubles to the ciliary membrane might play a role for the reversible bending of primary cilia in response to fluid flow, e.g., in kidney tubules [35].

### 2.3. Primary Ciliogenesis

Ciliogenesis and cell cycle are tightly connected with centrioles functioning as basal bodies for growing cilia. Primary ciliogenesis via the intracellular pathway (e.g., in RPE cells (Retinal Pigment Epithelial), fibroblasts and chondrocytes) is initiated near the nucleus by attaching presumably golgi-derived cytoplasmic vesicles to the mother centriole to form ciliary vesicles. The mother centriole then docks to the plasma membrane and releases the ciliary vesicles by exocytosis, exposing the budding cilia to the extracellular space, and a ciliary pocket is formed at the at the fusion site. The primary cilium then continues to grow from the tip until the predefined cell type specific length is reached and cilia are often submerged in the ciliary pocket [23,24]. However, kidney tubular cells and cholangiocytes use the alternative pathway. After cytokinesis, a protein complex rich of ciliary proteins named midbody remnant is formed and initiates ciliogenesis at the centrosome which locates at the apical cell surface in polarized cells. Since ciliogenesis is initiated at the plasma membrane, cilia lack the ciliary pocket, exposing cilia directly to the extracellular space, potentially enabling better detection of flow and other extracellular changes [36].

## 3. Dyneins

The term “Dynein force in protein” was coined by Gibbons and Rowe in 1965 after isolating ATPases similar to the mitotic spindle ATPases by density gradient fractionation of *Tetrahymena pyriformis* cilia [37,38]. The isolated giant motor protein complexes with >1-MDa molecular weight have since been scrutinized by cryo-EM (cryogenic electron microscopy) and Cryoelectron tomography techniques [39].

**Figure 2:**
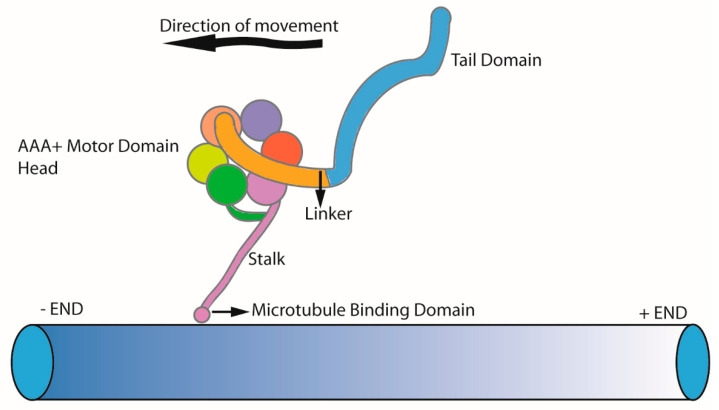
Simplified structure of the dynein heavy chain, containing a heavy chain head with an AAA+ motor domain, a stalk with a microtubule binding domain, a linker, and a variable tail domain where other dynein subunits bind.

Dyneins are minus-end directed ATPases moving along the microtubules. Dynein complex subunits are classified based on their molecular weight as heavy chains, intermediate chains, light intermediate chain, and light chain. Three main classes of dyneins can be distinguished: axonemal dyneins which power the bending motion of cilia; intra-flagellar transport dynein (IFT dynein or cytoplasmic dynein-2) driving the retrograde axonemal transport; and cytoplasmic dynein 1 which powers retrograde transport along cytoplasmic microtubules [40,41,42]. All dynein motors are built around a force generating a dynein heavy chain. The dynein heavy chain has a well conserved motor domain head with an AAA+ ring (ATPase associated with various cellular activities), a stalk which acts as a microtubule binding domain, a linker, and a variable tail domain where other dynein subunits binds. The interaction of the dynein heavy chain with other chains is important for its stability, cargo binding, and also for the coordinated movement [43]. This is highlighted by the fact that dynein-2 fails to homodimerize in the absence of intermediate chain and light chain subunits [39]. Specific cargos and adaptors are also attached to the tail domain, which leaves dynein motors well equipped to perform the specialized function in its operating environment (Figure 2) [44]. Sixteen genes encode for dynein heavy chains in humans, and the majority are associated with axonemal dyneins [41]. In this review, we focus on ciliary dynein motors IFT dynein (cytoplasmic dynein-2) and axonemal dyneins.

## 4. Intraflagellar Transport Dynein (IFT Dynein or Dynein-2)

### 4.1. IFT Dynein Function

Cytoplasmic dynein-2 or intraflagellar transport dynein (IFT dynein) is the motor for retrograde transport along the ciliary axoneme from the ciliary tip to the base. A complete loss of function of retrograde IFT results in stumpy cilia with a bulged tip and disorganized axoneme, while milder dysfunction manifests itself with the accumulation of proteins at the tip of the cilia [45,46,47]. IFT dynein also appears to play a role in assembling the transition zone components in primary cilia. In *Caenorhabditis elegans,* dysfunction of the IFT dynein heavy chain results in the accumulation of transition zone components in the distal end of the cilia [48]. Likewise, dynein-2 has been implicated in building a functional transition zone in human retinal pigment epithelium cells (RPE cells) [49].

Recent advances in electron microscopy techniques have paved the way for dissecting the dynein-2 structures to a near atomic level. Through time-resolved correlative fluorescence and three-dimensional electron microscopy in *Chlamydomonas* using GFP-tagged IFT cargos, Stepanek et al. illustrated the assignment of separate tracks for IFT trains moving at a speed of 2.5 to 4 µm/s. Anterograde trains use the B microtubule, while retrograde trains returning to the base travel on the A microtubule track [50]. Interestingly, the B microtubules seemed shorter and terminate earlier than the A microtubules, which extend to the tip of the cilium [35]. Dynein-2 is transported in an inactive form by kinesin motors on the B microtubule track from the ciliary base to towards the tip. During this process, the microtubule binding domain of the dynein motor is positioned away from the microtubules. At the ciliary tip, kinesin becomes phosphorylated, rendering it inactive, which in turn triggers conformational changes in the anterograde train. Dynein-2 then adopts an active conformation with the microtubule binding domain orienting towards the microtubules, enabling and functioning as a motor for the transport of ciliary components back to the base (minus end) of the cilium [51].

### 4.2. IFT Dynein Complex Structure

*Chlamydomonas* mutants lacking subunits of IFT dynein paved way for establishing the role of IFT dynein as a bona fide ciliary retrograde transport motor, and also facilitated understanding the structure of the complex. *Chlamydomonas* mutants for the heavy chain DHC1b show a defective retrograde transport and short flagella, indicating this heavy chain is essential for retrograde IFT in cilia [52]. In most organisms, two copies of the dynein heavy chain form a homodimer where the other subunits are attached [53], but in *Trypanosoma brucei,* two heavy chains encoded by two different genes form a heterodimer [54]. DYNC2H1 is the mammalian homologue of DHC1b [53]. Only one light intermediate chain associates with IFT dynein, named D1bLIC in *Chlamydomonas* [55] and DYNC2LI1 in mammals [56,57]. WDR34 [58,59] and WDR60 [60] are the two intermediate chains of the IFT dynein in humans, and their *Chlamydomonas* counter parts are termed D1bIC2 [61] and D1bIC1 [62], respectively. Light chains associated with the IFT dynein complex are divided into three groups, namely, the DYNLL/LC8, DYNLT/Tctex, and DYNLRB/LC7 groups [53].

In 2014, using protein interactome studies, Asante et al. showed that the light intermediate chain, DYNC2LI1, interacts with WDR34. They also observed the presence of light chains that were already proven to be associated with cytoplasmic dynein, like TCTEX-1 (DYNLT1), TCTEX-3 (DYNLT3), roadblock-1 (DYNLRB1), roadblock-2 (DYNLRB2), LC8-1 (DYNLL1), and LC8-2 (DYNLL2), in the WDR34 protein network. Additionally, they identified TCTEX1D2 as the unique IFT dynein specific light chain. WDR60 is a bigger protein compared with WDR34, and the association of these two intermediate heterodimeric complexes with other subunits in the complex is crucial for the function of IFT dynein [63]. In 2015, Gholkar et al. also ascertained the association of light chain TCTEX1D2 with WRD34 and WDR60 [64]. While *Chlamydomonas* only has two different dynein motors, axonemal dynein and cytoplasmic dynein acting in the cell body and along the flagellum, vertebrates have an additional cytoplasmic dynein motor, cytoplasmic dynein-1, present only in the cytosol, and the dysfunction of this motor results in neurological disorders.

**Figure 3:**
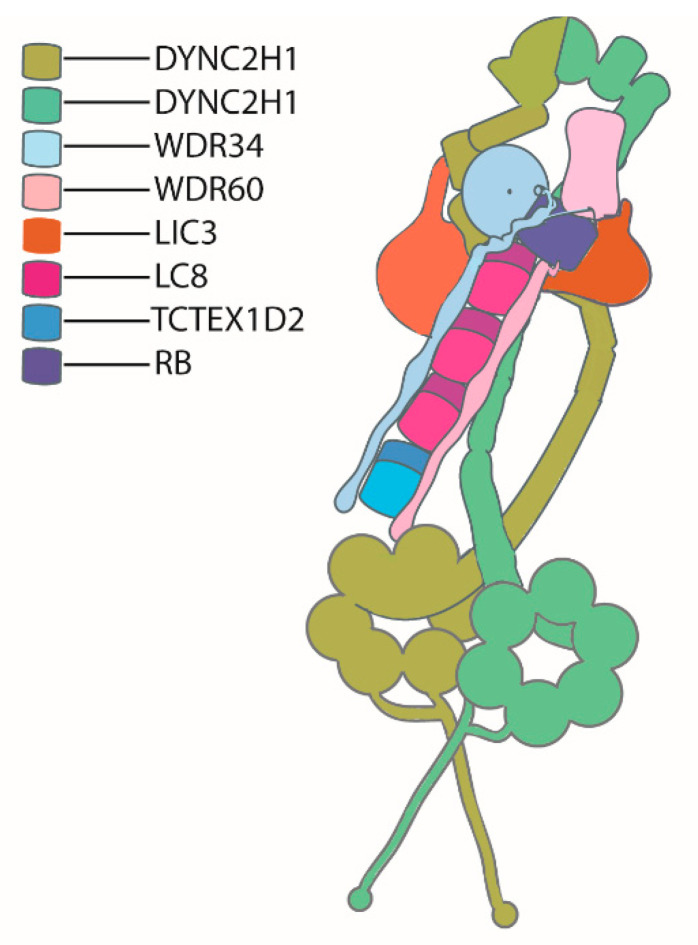
Sche matic of dynein-2, adapted from Toropova et al., 2019 [39]. Two copies of the dynein heavy chain form a homodimer, with one heavy chain adopting a zig-zag conformation at the tail region. One copy of the intermediate chain (DYNC2LI1 (LIC3)) is attached to each heavy chain. Two heterodimeric intermediate chains (WDR34 and WDR60) bind to DYNC2H1 via their *C*-terminal ends, and light chains are attached to the *N*-terminal ends of the intermediate chains.

Toropova et al. resolved the structure of human dynein-2 to a near atomic level using cryo-EM in 2019. They purified recombinantly expressed human dynein-2 complex (~1.4MDa). Dynein-2 has a sophisticated stoichiometry: two dynein heavy chain (DYNC2H1) molecules (possessing a condensed *N*-terminal tail domain and a *C*-terminal AAA+ motor domain) are present in each dynein-2 dimer, with one heavy chain adopting a zig zag conformation at the tail region. Each heavy chain binds a light intermediate chain (LIC3\DYNC2LI1). The two different intermediate chains, WDR34 and WDR60, form a heterodimer, binding DYNC2H1 via the beta propeller domain at the *C*-terminal end, while light chains are attached to the *N*-terminal domains. Light chains consist of three LC8 dimers, one roadblock (RB) dimer, and a TCTEX−TCTEX1D2 dimer. The zig-zag conformation of DYNC2H1 enables perfect locking in an inactive conformation during anterograde transport via IFT-B trains. The binding of the intermediate chain and light chain cluster (IC-LC cluster) with the heavy chain and light intermediate chain stabilizes the IFT dynein complex in its inhibited state for its anterograde transport, and upon reaching the tip IC-LC cluster, also aids in the confirmation change of the dynein motor for active retrograde transport [39]. A schematic of the presumed structure of dynein-2 is shown in Figure 3.

## 5. Motile Cilia

### 5.1. Motile Cilia Function

In mammals, motile cilia usually appear in bunches of 30–300 cilia on the epithelial cells lining the upper and lower respiratory tract, on brain ependymal cells, and on epithelial cells of the fallopian tubes. During mammalian embryonic development, motile cilia can also be found in the embryonic node [34,65]. Multiciliated cells in the respiratory tract are regenerated constantly, in contrast to ependymal cilia, which are solely formed during embryonic development and cannot be replenished [66,67]. The metachronal wave-like movement of these hundreds of cilia per cell can generate fluid flow and enable mucus transport [68,69]. Cerebrospinal fluid movement by ependymal cilia also helps in the distribution of signaling molecules and clearing of the toxins [66]. The ciliary beat frequency of the airway cilia reaches approximately 10–30 Hz [4,66,70]. Motile cilia on pit cells within the embryonic node generate a clockwise rotational movement [3] resulting in a leftward fluid flow, enabling the establishment of a signaling molecule gradient causing asymmetric gene expression and determining the left side of the organism [65]. Sperm cell flagella have a very similar architecture to motile cilia; however, they are much longer than motile cilia (50–60 µm) and move in a wave-like or sigmoidal fashion [12,69].

In addition to movements of fluids, motile cilia also have sensory functions. Motile cilia, for example, express TRPV4 channels, and the ciliary beat frequency is altered in response to calcium ion levels in the cytosol and extracellular space [71,72]. Microarray data of differentiated human airway epithelia cultures further suggest the expression of bitter taste receptors, and the introduction of bitter compounds can increase intracellular calcium, which in turn increases ciliary beat frequency, potentially so as to expel the harmful agent [73]. In addition, the membrane of the cilia lining in the fallopian tubes has a high density of progesterone, estrogen, and the interleukin-6 receptor. The receptor activation influences ciliary beat frequency, which facilitates egg transport and fertilization [74,75,76].

### 5.2. Motile Ciliogenesis

Motile ciliogenesis requires the inhibition of Notch signaling in multiciliated progenitor cells, enabling the differentiation of progenitor cells to multiciliated cells [77,78]. Reduction of BMP (bone morphogenetic protein) signaling has also been recently implicated in multiciliated cell differentiation [79,80]. Multiciliated cell differentiation is initiated by coiled coil domain containing GEMC1 (geminin coiled-coil containing protein 1) succeeded by MCIDAS/multicilin (multiciliate differentiation and DNA synthesis associated cell cycle protein) [78]. MCIDAS interacts with cell repressing transcription factors E2F4, E2F5, and DP1. This ternary complex regulates the expression of genes responsible for centriole generating, namely *PLK4*, *CEP152*, *STIL*, *DEUP1 (CCDC67)*, *MYB,* and *CCNO* [81,82,83]. Concurrently, major ciliary transcription factors (RFX 1, RFX 2, RF3, RFX 4 (Regulatory factor X), and FOXJ1 (Forkhead Box J1)) are upregulated [3,84,85]. While RFX factors activate the transcription of motile and non-motile ciliary genes, FOXJ1 is essential for the transcription of motility specific ciliary genes [3]. A recent study also showed that GEMC1 interacts with p73 and forms a complex with E2F5, subsequently activating transcription factors enabling multiciliated cell lineage differentiation centriole generation [86]. The activation of genes allowing generation of multiple centrioles is crucial, as these will act as basal bodies for motile cilia.

Two important pathways have been proposed for basal body biogenesis—the centriolar pathway and the deuterosome pathway. The centriolar pathway allows for less than 10 daughter centrioles to nucleate from the mother centriole [87]. The deuterosome pathway, in contrast, generates hundreds of centrioles. Deuterosomes consist of fibrogranular material located in the apical cytoplasm, and mature into centrioles, subsequently forming a basal root while the axoneme extension occurs from the apical surface [24,87]. *DEUP1* plays a key role in deuterosome mediated centriole amplification, whereas *CEP63* (Centrosomal Protein 63) drives the centriolar pathway [82]. The MICIDAS, E2F, and DP1 ternary complex drives deuterosome mediated centriole amplification [81]. Massive centriole biogenesis in multiciliated cells could also occur via a combination of both pathways [88] but interestingly, centriole formation in multiciliated cells seems to also occur in the absence of deuterosomes and parent centrioles, through centriole formation from the pericentriolar material, containing PCM1 protein and fibrogranular material [89]. FoxJ1 controls the basal body docking to the apical region of the plasma membrane, and multiple motile cilia are then grown from basal bodies by intraflagellar transport [90]. In summary, the complex molecular process underlying multiciliogenesis is not fully understood to date.

### 5.3. Motile Cilia Structure

**Figure 4:**
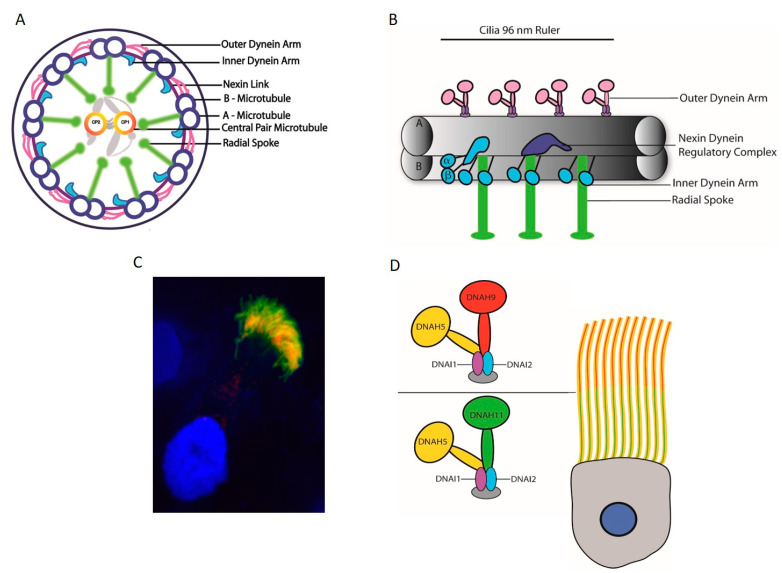
Schematic representation of motile cilia. (**A**) Motile cilia structure showing a 9 + 2 arrangement of microtubules, inner and outer dynein arms attached to the A microtubule, the nexin dynein regulatory complex (N-DRC) that links the A microtubule of one peripheral doublet to the B microtubule of the adjacent doublet, and a central pair with associated projections and radial spokes. (**B**) Four ODAs, one double headed IDA and six single-headed IDAs, accompanied by three radial spokes and one N-DRC protein complex, form a 96 nm ruler distributed along the axoneme of the motile cilia. (**C**) Immunofluorescence of motile respiratory cilia (green: DNAI1, red: acetylated tubulin, and blue: DAPI). (**D**) Outer dynein arm subtypes in human respiratory cilia. Two ODA subtypes defined by the localization of dynein heavy chains can be distinguished: DNAH11 localizes to the proximal half, while DNAH9 is found in the distal half of human respiratory cilia.

The axoneme of motile cilia is made up of a microtubule-based cytoskeleton arranged in an organized 9 + 2 pattern, with the exception of motile nodal cilia largely lacking central pair microtubules [34,91]. The sliding of the microtubules against each other enables ciliary motility and requires additional structural components, namely: inner and outer dynein arms, the outer dynein arm docking complex, the nexin dynein regulatory complex (N-DRC), and radial spokes (Figure 4A). N-DRC is a multiprotein complex that links the A microtubule of one peripheral doublet to the B microtubule of the neighboring doublet. Outer dynein arms (ODA) and inner dynein arms (IDA) are attached to the peripheral A microtubule and power the orchestrated movement of the cilia. The outer dynein arm docking complex enables the efficient docking of ODAs to the surface of the A microtubule. Radial spokes connect the peripheral microtubules to the central pair microtubules, and seem to serve as signaling centers. A central pair of microtubules consisting of thirteen protofilaments are connected by a bridge-like structure and have asymmetric projections at every 32 nm [92,93]. The interaction of these projections with radial spokes allows collision-based mechanosignaling, which is vital for the movement of dynein arms [94,95]. Along the axoneme, every 96 nm a repeat unit contains four ODAs, one double headed IDA, and six single headed IDAs, accompanied by three radial spokes and one N-DRC protein complex (96 nm ruler) (Figure 4B). As nodal cilia lack the central pair microtubules and radial spokes, the dynein arms enable a rotational instead of a wavelike movement [4,96].

## 6. Axonemal Dyneins

Dynein arms are central to ciliary movement and were first described by Afzelius in 1959 [97]. By fixing sea urchin spermatozoa in a new fixative, he observed protrusions from peripheral filaments, which he called “arms” [97]. The dynein arm function has been extensively studied in the green unicellular algae *Chlamydomonas reinhardtii,* and thanks to outstanding evolutionary conservation, the mechanistic findings of this multiprotein motor complex have since been transferred to vertebrates, including humans.

### 6.1. Axonemal Dynein Movement to Generate Force for Cilia Beating

Dynein arms are arranged around the peripheral microtubules, with their heavy chain tail domain attached to the A microtubule and a microtubule binding stalk positioned near the B microtubule of the nearby peripheral microtubule. Microtubule sliding is enabled by ATP hydrolysis at the AAA+ head domain of the heavy chain. The “switch point” model by Wais-Steider and Satir proposes that only half of the dynein is active during the active stroke, while the other half remains in an inactive conformation. The recovery stroke is created by switching the passive dynein arms to the active form, and vice versa. Organized repetition of this cycle creates a wave pattern movement [44,98]. More recently, the “switch inhibition” hypothesis by Lin and Nicastro, however, suggested that most dyneins are in their active form, as cryo-electron tomography in sea urchin sperm cells showed large angles between the linker and stalk of the dynein heavy chains. They proposed that where all dynein arms are active, dynein forces are equally distributed, keeping the flagella straight. During bend initiation at the flagella transition region, inner dynein arms adopt an inactive confirmation. The inactive confirmation of the inner dynein arms is then transferred to the corresponding outer dynein arm located in the same microtubule pair, exerting a counter force to the active dynein arms on the opposite side of the peripheral ring microtubules. This inhibition is switched efficiently between opposite sides, with less than 10 milliseconds to create a wave-like movement in the cilia and flagella. Hence, switching off the dynein arm activity spatiotemporally at regular intervals could generate metachronal ciliary movement [99]. Both models agree that the asymmetric distribution of the dynein motor activity at regularly spaced short intervals along the entire axoneme is an indispensable element for motile cilia movement.

### 6.2. Axonemal Motor Complexes

Outer and inner dynein arms project from the peripheral A microtubule, with outer dynein arm docking aided by the docking complex every 24 nm. Inner dynein arms along with three radial spokes and the N-DRC component are spaced at a distance of 96 nm. Inner dynein arms are divided into two groups—the first group with two heavy chains that form a double headed structure and the second group with one heavy chain. There is one double headed inner dynein arm, as well as six single headed inner dynein arms. The two headed inner dynein arm has three intermediate chains and four light chains, in contrast, only actin or centrin are identified as subunits of the single headed dynein arms [100]. In *Chlamydomonas*, the outer dynein arms (ODA) contain three different heavy chains (α, β, and γ), resulting in a three headed structure; along with these heavy chains, the complex also has two intermediate chains and nine light chains [101]. In humans, however, ODAs are double headed, with two types of heavy chains, two intermediate chains (DNAI1 and DNAI2), and several light chains [100,102]. Two different ODA subtypes, defined by the heavy chains DNAH9 and DNAH11, can be distinguished in human respiratory cilia (Figure 4C). ODA1 contains DNAH11, while ODA2 contains DNAH9, both of which are orthologous to the *Chlamydomonas* β heavy chain. While ODA1 is found only in the proximal half of the axoneme, ODA2 occurs only in the distal half (Figure 4D). DNAH5, orthologous to the *Chlamydomonas* γ heavy chain, is present in both ODA1 and ODA2. Interestingly, in sperm flagella, DNAH5 is only present in the proximal flagella region, and DNAH9 in turn shows a panaxonemal distribution [103,104]. The outer dynein arms generate the force driving ciliary movement. The inner dynein arms are responsible for creating the discrete ciliary wave pattern by controlling the size and shape of the ciliary bend. Along with the inner dynein arms, radial spokes and nexin Dynein regulatory complexes (N-DRCs) play an important role in regulating metachronal wave formation in cilia [105]. Dynein arms are assembled in the cytoplasm by dynein preassembly factors [106] and are transported to the ciliary axoneme by IFT cargo [47].

## 7. Ciliopathies

As ubiquitous structures, cilia play a central role in the development and survival of organisms. Defects in ciliogenesis, as well as in the ciliary structure and function, cause a diverse range of heterogeneous multisystem disorders with variable phenotypes and high mortality, termed “ciliopathies”. Most ciliopathies are very rare conditions affecting less than 1 in 2000 individuals, often less than 1:100,0000 with the exception of autosomal dominant polycystic kidney disease, which affects up to 1 in 1000–2000 persons [4,107]. Over 30 different ciliopathies have been identified, with more than 200 associated genes [108,109,110]. Primary ciliary dysfunction can cause defects in sensory and visceral organs, including anosmia [111], hearing loss [112], retinal degeneration [29], polycystic kidney disease [113,114,115], nephronophthisis [116], renal dysplasias [117], congenital fibrocystic diseases [118], polydactyly, short bones [119], and primary cilia have been also linked in the development of certain forms of cancer [104,120]. Motile cilia dysfunction can result in recurrent airway infections, laterality disorders, fertility issues, and hydrocephalus [121,122].

Ciliopathies are monogenic disorders most often following an autosomal-recessive inheritance pattern; more rarely, X-chromosomal, and very rarely, autosomal-dominant, inheritance patterns are observed. Interestingly, for non-motile ciliopathies, mutations in the same genes result in phenotypic heterogeneity, to the extent that entirely different disease entities can be observed [123]. For example, mutations in *WDR35* can cause short rib-polydactyly syndromes and Sensenbrenner syndrome [119,123], and mutations in several genes can cause nephronophthisis, Joubert syndrome, Meckel syndrome, and Bardet-Biedl syndrome [124]. At the same time, overlapping phenotypes are noticed in different ciliopathies, for example, situs inversus is observed in primary ciliary dyskinesia, Bardet-Biedl syndrome, and Joubert syndrome [121,125,126]. Similarly, obesity is commonly seen in Bardet-Biedl syndrome and Alstrom syndrome [123]. Over 100 genes have been implicated in ciliopathies to date, imposing additional diagnostic challenges [124].

Dynein related ciliopathies can be subdivided into two groups: IFT dynein dysfunction, which causes a group of non-motile cilia-based phenotypes summarized under short rib thoracic dysplasias, and axonemal dynein dysfunction, representing the most common cause for the ciliary motility defect, also commonly known as primary ciliary dyskinesia (PCD).

### 7.1. IFT Dynein Related Ciliopathies

**Figure 5:**
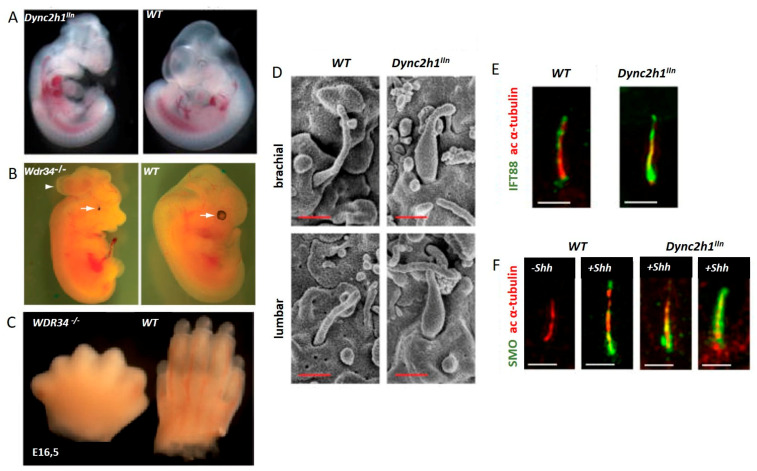
IFT dynein dysfunction results in a complex developmental phenotype in mice. (**A**) *Dync2h1* loss of function mouse embryo showing a turning defect and CNS defects compared to a control embryo, reprinted with permission from Ocbina et al. Nat Genet 2011 Jun;43(6):547-53 [46]. (**B**,**C**) *Wdr34* mutant mouse embryos compared to controls, reprinted with permission from Wu et al. Hum Mol Genet. 2017 Jul 1;26(13):2386–2397 [128]. White arrows in (**B**) indicate microphthalmia *Wdr34* mutant embryo compared to control; the arrow head indicates encephalocele in the *Wdr34* mutant (**D**) Shortened stumpy primary cilia in *Dync2h1* mutant mice compared to controls, visualized using electron microscopy. (**E**) Accumulation of IFT 88 within the stumpy *Dync2h1* mutant cilia compared to controls. (**F**) Ciliary accumulation of Smo in *Dynch1* dysfunctional cells in the absence of Shh, as well as after Shh stimulation, compared with control cells, reprinted with permission from Ocbina et al. Nat Genet 2011 Jun;43(6):547-53 [46].

*Chlamydomonas* with dynein heavy chain defects have short flagella filled with abnormal microtubules and cargo (rafts) [52,127]. Both *DYNC2H1* and *WDR34* null mutant mice embryos do not survive beyond mid-gestation. The cause of death is not clear, however neural tube defects including open brain and spinal bifida, shortened bones, and polydactyly can be observed. Likewise, the accumulation of IFT components in the cilia, as well as Hedgehog (Hh) signaling defects, occur in both models [46,128] (Figure 5).

No human patients with biallelic null variants in *DYNC2H1* or *WDR34* have been identified to date, likely because of embryonic lethality; all reported cases carry at least one presumably hypomorphic missense allele [58,60,129,130,131,132,133]. In humans, impaired retrograde IFT motor dysfunction results in a complex developmental, often lethal, phenotype in humans summarized under the term short-rib thoracic dysplasia, with or without polydactyly (OMIM #611263, #613091, #263520, # 269860, # 614091, and #208500). Historically, these phenotypes were also named short rib polydactyly syndrome (SRPS) or Jeune asphyxiating thoracic dystrophy (JATD)/Jeune syndrome. SRPS can be considered the severe end of the spectrum here, defined as lethal in utero or during the neonatal period, while survival beyond the neonatal period is possible although not always achieved for JATD cases.

**Figure 6:**
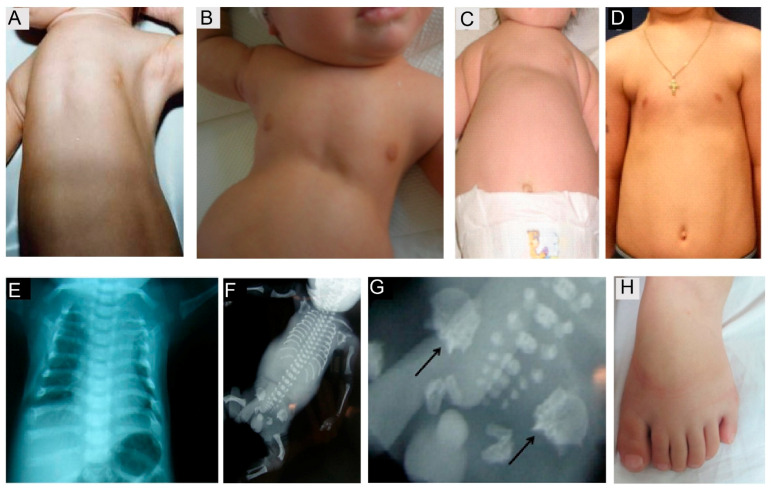
Skeletal features observed in short rib polydactyly syndrome patients. The main hallmark is thoracic narrowing present, observed in utero or from birth (**A**–**C**), becoming less pronounced with increasing age (**D**), (**E**) short horizontal ribs in a JATD case SRPS case with shortened long bones, long narrow thorax, pelvis configuration with acetabular spurs (**F**, close up in **G**, arrows indicate spurs). JATD case with a narrow thorax and shortened ribs, as well as handlebar clavicles, shown in the thorax X-ray, (**A**) reprinted with permission from Schmidts et al. J Med Genet 2013 May;50(5):309-23 [135], (**B**) reprinted with permission from Halbritter et al. Am J Hum Genet 2013 *93*, 915–925 [136], (**C**–**E**) reprinted with permission from Schmidts et al. Am J Hum Genet. 2013 Nov 7;93(5):932–944 [131]; (**F**,**G**) reprinted with permission from Mc Inerney-Leo et al. Am J Hum Genet 2013 Sep 5;93(3):515-23 [133]. Polydactyly (**H**) is more frequently observed in SRPS compared with JATD, reprinted with permission from Schmidts et al. Nat Commun. 2015 Jun 5;6: 7074 [133].

SRPS and JATD represent so-called ciliary chondrodysplasias, with overlapping skeletal and extra-skeletal presentations. The main hallmarks are short ribs, resulting in a narrowed bell shaped thorax as and variable shortening of the long bones. Thoracic size is life limiting in the first two years of life, resulting in respiratory insufficiency due to pulmonary hypoplasia. Further radiological signs include a trident acetabulum with spurs in some forms of SRPS and JATD, as well as cone-shaped epiphyses in older JATD patients. Polydactyly is often observed in SRPS, but more rarely in Jeune syndrome (Figure 6). Nephronophthisis (NPHP) and, more rarely, renal cysts can be observed with JATD and SRPS. Likewise, retinal degeneration occurs in some forms of Jeune Syndrome, especially in individuals with NPHP, while SRPS patients do not survive long enough to develop retinal symptoms. Furthermore, cardiac and gastrointestinal defects can occur in SRPS. In Jeune syndrome, altered liver enzymes are observed frequently; however, visible liver cysts are rarely observed, while liver cyst formation and liver fibrosis are more frequently found in the autopsies of SRPS cases [134].

**Figure 7:**
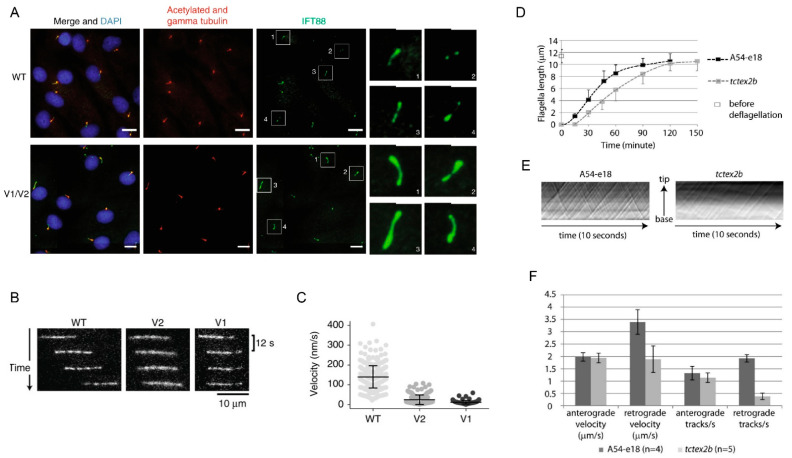
Ciliary defects observed in IFT dynein patients. (**A**) Bulged ciliary tips and accumulation of IFT particles at the ciliary tips in fibroblast cilia from an individual carrying *DYNC2H1* mutations; enlarged images of the cilia marked in the white squares are shown on the right side with pictures numbered accordingly and (**B**,**C**) reduced tubulin velocity compared with the control, indicating impaired IFT, reprinted with permission from Vig et al. Genet Med. 2020 Dec;22(12):2041–2051 [137]. (**D**) Slower flagella extension, but similar flagella end length in *Tctex2b* mutant *Chlamydomonas* compared with controls suggests partial functional redundancy for Tctex2b. (**E**) Reduced but not absent retrograde IFT in *Tctex2b* mutants compared with controls. (**F**) Normal anterograde IFT velocity and unchanged number of anterograde IFT trains, but a reduction of the retrograde velocity and a strong reduction in the number of retrograde IFT trains in *Tctex2b* mutant *Chlamydomonas* compared with controls, reprinted with permission from Schmidts et al. Nat Commun. 2015 Jun 5;6: 7074 [133].

Most SRPS and JATD cases result from mutations in genes encoding for dynein-2 components: the heavy chain *DYNC2H1* [130], intermediate chains *WDR34* [58,131] and *WDR60* [60], the light intermediate chain *DYNC2LI1* [132], and the light chain *TCTEX1D2* [133]. Overall, the vast majority of human SRPS/JATD cases are caused by altered DYNC2H1 function, likely because *DYNC2H1* is a very large gene [135]. *WDR34* mutations also cause a significant number of cases, while variants in *WDR60, DYNC2LI1,* and *TCTEX1D2* are rarely observed. Interestingly, no causative variants in other light chains such as LC8 or RB have been identified to date. In accordance with the role of IFT dynein as a motor for retrograde IFT, patient fibroblasts display an accumulation of IFT components at the ciliary tip (Figure 7). Recently, biallelic *DYNC2H1* full loss of function mutations affecting the retina specific transcript have been identified in individuals with isolated retinal degeneration without any other ciliopathy phenotypes [137]. Interestingly, patient fibroblasts showed a normal ciliation but IFT88 accumulation at the ciliary tip, similar to what is observed in JATD individuals (Figure 7).

In contrast with individuals carrying biallelic mutations in genes encoding other IFT dynein components, individuals with JATD carrying biallelic *TCTEX1D2* mutations may represent true nulls. However, patient fibroblasts show a similar degree of IFT88 accumulation as observed in the fibroblasts from presumably hypomorphic patients with *DYNC2H1* mutations, but still form cilia, suggesting TCTEX1D2 is at least partially redundant for IFT dynein function [133]. This is supported by the observation that retrograde IFT is only slowed down, but not stopped in *Chlamydomonas* deficient for the TCTEX1D2 homologue (Figure 7). Mutations in *NEK1*, a gene encoding for a component localizing to the ciliary base, as well as the IFT-A component *WDR35,* have likewise been described in SRPS cases [138,139].

Causative *DYNC2LI1* variants have further been linked to Ellis-van Creveld syndrome, a ciliary chondrodysplasia with additional ectodermal and cardiac defects [140]. *WDR34* mutations can also cause non-syndromic rod−cone dystrophy (a single case reported) [141]. In contrast with JATD patients with IFT-gene mutation related ciliopathies, patients with dynein gene mutations show a more severe rib and lung phenotypes, often leading to neonatal death [129,135,136,142]. However, the surviving patients usually do not seem to develop clinically relevant eye or kidney disease until mid-adulthood; however, because of the low number of adult patients reported, it is unclear if this may occur later in life [129,135]. In contrast, IFT gene mutations seem to cause a mild rib phenotype, but nearly all JATD patients with IFT gene variants develop renal and retinal degeneration during childhood or adolescence [129,142]. The pathomechanism behind this observation has remained elusive.

In vertebrates, Hh signaling is important for tissue homeostasis and organ development. Three types of hedgehog proteins, namely Indian hedgehog (Ihh), Sonic hedgehog (Shh), and Desert hedgehog (Dhh), play a crucial role in the development of vertebrates [143]. Dhh is important for the development of the testis and ovaries [144,145]. Central nervous system development, limb patterning, and the determination of digits are controlled by Shh [143,146]. Ihh is essential for chondrocyte proliferation, maturation, and osteoblast development, hence playing a vital role in skeletal development [147]. The Hh signaling receptor patched locates to the primary cilia and inhibits the movement of smoothened to cilia when it lacks Hh ligand binding. In the absence of smoothened GLI transcription factors, GLI 2 and GLI 3 are cleaved and degraded by the ubiquitin-proteasome pathway. Hh binding causes smoothened ciliary entry and the GLIs can activate gene transcription [27,148,149]. In human ciliary chondrodysplasia patients and IFT dynein mutant mouse models, the accumulation of hedgehog signaling components has been observed, including Gli2 accumulation at the base of the stumpy cilia in WDR60 patient fibroblasts and SMO accumulated in cilia of *Dync2h1* mutant mouse model [46,60] (Figure 5).

### 7.2. Axonemal Dynein Related Ciliopathies

**Figure 8:**
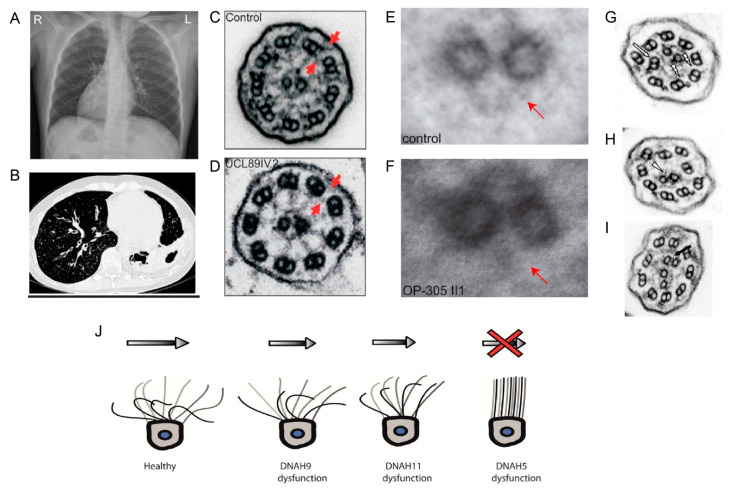
The clinical phenotype of PCD and the representative EM defects observed. (**A**) Chest X ray of patient showing situs inversus, reprinted with permission from Loges et al. Am J Hum Genet. 2018 Dec 6; 103 (6):995–1008 [158]. (**B**) PCD patient with bronchiectasis of the right and left lower lobes, reprinted with permission from Onoufriadis et al. Am J Hum Genet. 2013 Jan 10; 92(1):88–98 [150]. (**C**–**I**) Cross section of the cilia with ultra-structural defects in comparison with the control. (**C**) Control with outer and inner dynein arms (red arrows); (**D**) DNAAF3 patient’s cilia lacking both outer and inner dynein arms (arrows), reprinted with permission from Mitchison et al. Nat Genet. 2012 Mar 4;44(4):381–389 [159]. (**E**) Control with central pair appendage (arrow) and (**F**) Hydin patient cilia with missing central pair appendage (arrow) visualized by image averaging, reprinted with permission from Olbrich et al. Am J Hum Genet. 2012 Oct 5; 91(4):672–684 [160] (**G**) disorganized peripheral microtubules and (**H**) acentric central pair, as well as (**I**) supernumerary central pairs seen in the cilia of CCDC40 patients, reprinted with permission from Antony et al.Hum Mutat. 2013 Mar; 34(3):462–472 [161]. (**J**) Graphical demonstration of a normal motile cilia movement pattern compared with the pattern observed in DNAH9 dysfunction (immotile distal half), DNAH11 dysfunction (immotile proximal half), and DNAH5 dysfunction (complete immobility), with recovery strokes shown in black.

Axonemal dynein related ciliopathies are caused by impairment of the coordinated movement of cilia. These conditions are summarized under the term primary ciliary dyskinesia (PCD) (OMIM #244400). PCD is a chronic respiratory disease occurring at a frequency of approximately 1 in 10,000−20,000 births [121]. Defective mucus and pathogen transport out of the airways due to impaired cilia motility causes recurrent infections, subsequently damaging the respiratory tract tissues. Neonatal respiratory insufficiency, chronic or recurrent sinusitis, middle ear infections, and upper and lower airway infections including pneumonia, resulting in progressive lung damage and bronchiectasis are classical symptoms [150]. Lung failure can occur in advanced adulthood. Subfertility due to dysmotility of the sperm flagella in males and fallopian tube cilia in females is also frequently observed [4,151]. Approximately half of all PCD patients present with laterality defects [152,153,154] (Figure 8), including a complete mirror image of the normal situs (situs inversus) or random arrangement (situs ambiguous or heterotaxy) of thoracoabdominal organs [153,154,155]. While organ function is not usually compromised with situs inversus, heterotaxy is associated with a higher rate of congenital heart defects, including transposition of the great arteries [154]. Laterality defects can further present as left- or right-isomerism, resulting in polysplenia or asplenia [156]. PCD plus situs inversus is also referred to as Kartagener’s syndrome [157].

The first proof that ciliary and flagella beating depends on outer dynein arms dates back to 1973, when B. H. Gibbons and I. R. Gibbons extracted the sea urchin sperm and found that removing outer dynein arms reduced flagella motility and showed that the beat frequency reduction was directly proportional to the number of arms removed [162]. Sea urchin studies were followed by investigations of *Chlamydomonas,* laying the indispensable basis for future human PCD studies. Studies on these single celled biflagellate also contributed tremendously towards deciphering the structure and function of primary cilia. In 1985, Kamiya et al. emphasized the importance of ODAs in creating flagellar waveform movement using the *Chlamydomonas Oda38* (*Oda1*) mutant, which lacked outer dynein arms. *Oda38* mutants displayed only a subtle change in the flagellar beta amplitude, however when stimulated with intense light, the mutants did not swim backward, in contrast with wildtype algae [163]. An *Oda1* follow up study from 1987 using a range of outer dynein arm mutants (*Oda38, Oda41, pfl3A,* and *supPfl*) and inner dynein arm mutants (*ida98* and *pf30*) further confirmed that outer dynein arms are critical for flagellar beat frequency, with a greater reduction of the beat frequency observed in ODA mutants, but IDA mutants showing a reduction in the wave amplitude, affecting the sliding velocity [164]. *Ic78*, the intermediate chain *Chlamydomonas* mutant, had missing outer dynein arms and showed slow irregular movement [165]. A candidate gene screen based on this mutant led to the identification of its human homologue *DNAI1* and to the association of this gene with PCD [166]. *Oda2* mutant *Chlamydomonas* facilitated the identification of the *DNAH5* locus in PCD patients with missing outer dynein arms [167]. *Pf22*, a dynein assembly factor *Chlamydomonas* mutant that was non-motile, with short flagella, ODA assembly defects, and also with missing inner dynein arms, assisted in proving the association of human *DNAAF3* with PCD [159].

To date, genetic defects in more than 40 genes have been found to cause PCD or mucociliary clearance disorders (Table 1), explaining 50−80% of the cases, depending on the investigated population [168,169]. PCD can be caused by defects in any of the motility apparatus component (Figure 4); however, dynein arm defects account for the majority of cases [169]. Among the PCD genes identified to date, 12 genes cause only ODA defects, disrupting the ODA complex or by inhibiting the attachment of the outer dynein arm to the microtubules. In contrast, 11 genes that encode the dynein assembly factor, as well as *CCDC103*, a dynein arm attachment gene, show defects in both dynein arms [169] while the term primary ciliary dyskinesia originates from observations of dyskinetic cilia in patients with dynein arm defects [169,170]. Ciliary motility defects and ultra-structural defects are variable, depending on the underlying gene defect. Mutations in the components of the same complex can cause different beating defects, depending on the severity of the loss of function defect. For example, mutations in *DNAH5*, an ODA heavy chain, cause immotile cilia [171]. However, the loss of function of NME8, an ODA intermediate chain, does not change the ciliary beat frequency significantly [172]. The loss of function of DNAH9 affects the distal localization of ODA components with defective distal movement of the cilia. In contrast, DNAH11 loss of function results in, defective proximal ODA subunits and proximal ciliary movement impairment [158] [103,104]. Mutations in ODA docking complex genes usually cause a complete absence of outer dynein arms and static cilia [168]. Mutation in *CFAP57,* an inner arm assembly component, causes a mild respiratory phenotype, where ciliary movement shows heterogeneous waveforms with reduced frequency, and patients do not show any defect in the ultra-structure of the cilia [173]. PCD patients with only inner dynein arm defects are difficult to diagnose; often, repeat collection of samples for EM and ciliary beat pattern analysis is required [174]. A lack of function of several different dynein assembly factors causes the absence of inner and outer dynein arms in respiratory cilia, resulting in a static cilia phenotype in this cohort of PCD patients [168]. Mutations in the axonemal 96 nm rulers *CCDC39* and *CCDC40* result in a disorganized arrangement of microtubules, reduction or absence of inner dynein arms, and defective radial spokes, and nexin links patients have non-coordinated movement of the cilia [161,175,176]. The absence of C2b projections of the central pair observed in patients with *HYDIN* mutations can be visualized only by EM tomography, and ciliary movement defects due to loss of HYDIN are subtle, causing diagnostic difficulties [160]. Representative EM images of ciliary defects observed in axonemal dynein related ciliopathy are given in Figure 8.

Embryonic nodal cilia differ from other motile cilia, as they lack the central pair and therefore also radial spokes. The embryonic node is the main left-right organizing center in mammals, including humans; hence, mutations in the genes encoding central pair or radial spoke components result in a PCD phenotype, but no laterality defects. Vice versa, laterality defects can also be caused by mutations in genes unrelated to cilia motility, but instead encoding for components of sensory cilia or signaling pathways crucial for left-right patterning such as *PKD2*, *NPHP*, *ZIC3*, and *NODAL* [156]. Heterotaxy can result in complex heart defects requiring extensive surgical measures, leading to symptoms which may cover up an underlying PCD phenotype [153,155]. Unfortunately, by far not all laterality defect patients are investigated for PCD, and it is likely that many PCD cases are missed. This is tragic, as the early start of supportive therapy such as antibiotics, inhalation, and physiotherapy can slow down lung damage progression dramatically.

Mouse mutants of both dynein heavy chains *DNAH5* and *DNAH11* show heterotaxy and congenital heart defects. *DNAH5* mutant mice also present with reduced ependymal flow and frequently develop hydrocephalus [177,178,179]. Interestingly, *DNAH11* mutant mice have immotile respiratory cilia in contrast to humans, where cilia motility is not entirely abrogated but a rather stiff high frequency beating pattern can be observed [103,180]. Likewise, while the majority of PCD mouse models develop hydrocephalus causing high lethality, human PCD patients rarely do [181].

Air−liquid interphase culture of primary epithelial cells from PCD patients are a useful additional tool to study the ciliary beating pattern and ciliary structure abnormalities aseffects caused by recent viral or bacterial infections mimicking PCD confounding PCD diagnostics can be excluded [182].

PCD diagnosis is complicated, and ideally should be based on a combination of several different tests, namely: low nasal nitric oxide (nNO) levels (<77 nL/min) [183], electron micrograph (EM) of ciliary sections, and high speed video microscopy (HSVM) recordings of ciliary movement [184]. Nonetheless, the practice of using EM and HSVM is impeded by the requirement of specialized microscopes. In addition, approximately 30% of PCD patients show a normal ultrastructure in EM [185]. Diagnosis should therefore be complemented by the immunofluorescence analysis of the nasal motile cilia in order to visualize potentially missing motility components or demonstrate mislocalization [186]. This is particularly helpful in cases with subtle motility defects in video microscopy or normal EM findings. As a gold standard, PCD diagnostics should also contain genetic analysis. Genetic screening is particularly helpful to secure a PCD diagnosis in cases with subtle or no motility or EM defects, as well as generally to classify the underlying molecular defect.

## 8. Conclusions and Future Prospects

Enabling fluid propelling by motile cilia movement and cargo transport in non-motile cilia, dynein motors power the development, survival, and wellbeing of human beings. Hence, understanding the structure and function of these ciliary motors has made a great impact on understanding vertebrate development and human ciliopathy diseases. While the genetic basis of IFT dynein related conditions is clear by now, there will likely be additional axonemal dynein defects to be unraveled in the future, aiding in a swift diagnosis for PCD patients. For IFT dynein defects, the neonatal or intra uterine death of the affected human individuals hinders the availability of the patient material necessary for studying the pathomechanism of the phenotype. Recreating hypomorphic patient alleles in cell models could help in delineating the molecular mechanism underlying this disease. Ameliorating ciliary dynein defects is an ultimate aim—here, gene therapy approaches and pharmacological approaches could hold promising outlooks.

## Figures and Tables

**Table 1:** Known PCD genes with ciliary defects observed.

Sl. No	Gene Name	Protein Function	EM Description and Beat Pattern of Respiratory Cilia	Laterality Defects	OMIM
1	*DNAH5*	ODA Heavy Chain	Absence of ODAs Immotile cilia	yes	603335
2	*DNAH11*	ODA Heavy Chain	Partial reduction of ODAs in the proximal region Stiffness and reduced beating in the proximal region of the cilia	yes	603339
3	*DNAH9*	ODA Heavy Chain	Partial reduction of ODAs in the distal region Impaired ciliary bending at the distal end the of cilia	yes	603330
4	*DNAI1*	ODA Intermediate Chain	Absence or truncated ODAs Immotile cilia	yes	604366
5	*DNAI2*	ODA Intermediate Chain	Absence of ODAs Minimal residual motility	yes	605483
6	*DNAL1*	ODA Light chain	Absence of ODAs Reduced/absent motility	yes	610062
7	*NME8*	ODA Intermediate Chain	Shortened or absent ODAs Normal beat frequency	yes	607421
8	*CCDC114*	ODA docking complex	Absence of ODAs Large areas of static cilia with 1–2 cilia showing stiff movement	yes	615038
9	*ARMC4*	ODA docking complex	Reduction of ODAs Reduce beat frequency and amplitude or static cilia	yes	615408
10	*CCDC151*	ODA docking complex	Absence of ODAs Static cilia	yes	615956
11	*TTC25*	ODA docking complex	Absence of ODAs Static cilia	yes	617095
12	MNS1 *	ODA docking complex	Slight reduction of ODAs Ciliary motility is not altered significantly	Yes	610766
13	*CCDC103*	Dynein arm attachment	Absent or defective ODAs and IDAs Static cilia	yes	614677
14	*DNAAF1*	Dynein preassembly factor	Absent or defective ODAs and IDAs Static cilia	yes	613190
15	*DNAAF2*	Dynein preassembly factor	Absent or defective ODAs and IDAs Static cilia	yes	612517
16	*DNAAF3*	Dynein preassembly factor	Absent or defective ODAs and IDAs Static cilia	yes	614566
17	*DNAAF4*	Dynein preassembly factor	Absent or defective ODAs and IDAs Static cilia	yes	608706
18	*DNAAF5*	Dynein preassembly factor	Absent or defective ODAs and IDAs Static cilia	yes	614864
19	*LRRC6*	Dynein preassembly factor	Absent or defective ODAs and IDAs Static cilia	yes	614930
20	*ZMYND10*	Dynein preassembly factor	Absent of ODAs and IDAs Static cilia	yes	607070
21	*SPAG1*	Dynein preassembly factor	Absent or defective ODAs and IDAs Static cilia	yes	603395
22	*C21ORF59*	Dynein preassembly factor	Absent or defective ODAs and IDAs Static cilia	yes	615494
23	*PIH1D3*	Dynein preassembly factor	Absent or defective ODAs and IDAs Static cilia	yes	300933
24	*CFAP300*	Dynein preassembly factor	Absent or defective ODAs and IDAs Static cilia	yes	618058
25	*CCDC164*	Nexin Dynein Regulatory Complex (N-DRC)	Missing N-DRC links Reduced amplitude and stiff compared with the control	no	615288
26	*CCDC65*	Nexin Dynein Regulatory Complex (N-DRC)	Normal ODAs, reduction in IDAs and nexin links, and MT disorganization Dyskinetic or hyperkinetic cilia	no	611088
27	*GAS8*	Nexin Dynein Regulatory Complex (N-DRC)	Misaligned outer doublet MTs Subtle reduction in the beating amplitude compared with the control	no	605178
28	*CCDC39*	96-nm Axonemal ruler	Axonemal disorganization with mislocalized peripheral MTs, absence of the central pair, supernumerary central pairs, reduction or absence of inner dynein arms, and defective radial spokes and nexin links Reduced amplitude with rigid axonemes that showed fast, flickery movements and defective beat regulation	yes	613798
29	*CCDC40*	96-nm Axonemal ruler	Axonemal disorganization with mislocalized peripheral MTs, absence of the central pair, supernumerary central pairs, reduction or absence of inner dynein arms, and defective radial spokes and nexin links Reduced amplitude with rigid axonemes that showed fast, flickery movements and defective beat regulation	yes	613799
30	*CFAP57*	Important for assembly of IDAs	Normal EM Heterogeneous waveforms in ciliary movement with reduced frequency	Single case, no laterality defect reported	614259
31	*MCIDAS*	Transcription factor	Reduced number of cilia and basal bodies, and immotile cilia	No	614086
32	*FOXJ1*	Motile ciliogenesis	Reduced number of cilia, and mislocalized basal bodies Stiff beating pattern with a reduced beat amplitude	yes	602291
33	*CCNO*	Amplification of centrioles	Reduced number of cilia and the few cilia present are motile	No	607752
34	*RSPH1*	Radial spoke head	Central complex and radial spoke abnormalities, different ciliary beating patterns (cilia with a normal beat frequency combined with abnormal motion, immotile cilia and cilia with a slowed beat frequency)	No	609314
35	*RSPH4A*	Radial spoke head	Central complex abnormality and abnormal circular movement	No	612647
36	*RSPH9*	Radial spoke head	Central complex abnormality and abnormal circular movement	No	612648
37	*RSPH3*	Radial spoke stalk	Central complex abnormality, absence of radial spokes, coexistence of immotile cilia, and motile cilia with movements of a reduced amplitude	No	615876
38	*DNAJB13*	Radial spoke neck	Central complex abnormality, lower beating frequency with reduced amplitude	No	610263
39	*HYDIN*	Central apparatus	Lacking C2b projection of the central pair, central pair number abnormality, and reduced beating amplitudes	No	610812
40	*STK36*	Between central pair and radial spoke	Central complex and basal foot abnormality, lacked coordinated beating, and stiff beating pattern with a reduced amplitude	No	607652
41	*SPEF2*	Central apparatus	Central complex abnormality and reduced beating amplitudes	No	610172
42	*CFAP221*	Central pair projection component	Normal ciliary structure and reduced beat frequency with abnormal circular movement	No	618704
43	*RPGR*	Transitional zone	Mixture of motile and immotile cilia and absence of various axonemal structures (dynein arms, the central pair, and nexin links)	No	312610
44	*OFD1*	Basal body	Normal ciliary structure Impaired ciliary motility	No	300170
45	*GAS2L2*	Basal body, basal feet, rootlets, and actin filaments	Normal ciliary structure, and defects in the orientation of cilia with asynchronous and hyperkinetic beat patterns	No	611398
46	*LRRC56*	Intraflagellar transport	Normal ciliary structure and severely dyskinetic cilia	Yes	618227

*—laterality defect and male infertility, no PCD; ODAs—outer dynein arms; IDAs—inner dynein arms; EM—electron microscopy; NA—not available.

## Data Availability

Data is available on personal request.

## References

[1] Satir P. (1995). Landmarks in cilia research from leeuwenhoek to US. Cell Motil. Cytoskelet..

[2] Elliott K.H., Brugmann S.A. (2019). Sending mixed signals: Cilia-dependent signaling during development and disease. Dev. Biol..

[3] Choksi S.P., Lauter G., Swoboda P., Roy S. (2014). Switching on cilia: Transcriptional networks regulating ciliogene-sis. Development.

[4] Mitchison H.M., Valente E.M. (2016). Motile and non-motile cilia in human pathology: From function to phenotypes. J. Pathol..

[5] Sironen A., Shoemark A., Patel M., Loebinger M.R., Mitchison H.M. (2020). Sperm defects in primary ciliary dyskinesia and related causes of male infertility. Cell Mol. Life Sci..

[6] Sagan L. (1967). On the origin of mitosing cells. J. Theor. Biol..

[7] Cavalier-Smith T. (1978). The evolutionary origin and phylogeny of microtubules, mitotic spindles and eukaryote flagella. Biosystems.

[8] Satir P., Guerra C., Bell A.J. (2007). Evolution and persistence of the cilium. Cell Motil. Cytoskelet..

[9] Vincensini L., Blisnick T., Bastin P. (2011). 1001 model organisms to study cilia and flagella. Biol. Cell.

[10] Malicki J.J., Johnson C.A. (2017). The Cilium: Cellular Antenna and Central Processing Unit. Trends Cell Biol..

[11] Ishikawa T. (2016). Axoneme Structure from Motile Cilia. Cold Spring Harb. Perspect. Biol..

[12] Kempeneers C., Chilvers M.A. (2018). To beat, or not to beat, that is question! The spectrum of ciliopathies. Pediatr. Pulmonol..

[13] Behnke O., Forer A. (1967). Evidence for Four Classes of Microtubules in Individual Cells. J. Cell Sci..

[14] Wloga D., Gaertig J. (2010). Post-translational modifications of microtubules. J. Cell Sci..

[15] Orbach R., Howard J. (2019). The dynamic and structural properties of axonemal tubulins support the high length stability of cilia. Nat. Commun..

[16] Kozminski K.G., Johnson K.A., Forscher P., Rosenbaum J.L. (1993). A motility in the eukaryotic flagellum unrelated to flagellar beating. Proc. Natl. Acad. Sci. USA.

[17] Ishikawa H., Marshall W.F. (2017). Intraflagellar Transport and Ciliary Dynamics. Cold Spring Harb. Perspect. Biol..

[18] Pedersen L.B., Rosenbaum J.L. (2008). Intraflagellar transport (ift) role in ciliary assembly, resorption and signalling. Curr. Top. Dev. Biol..

[19] Prevo B., Scholey J.M., Peterman E.J.G. (2017). Intraflagellar transport: Mechanisms of motor action, cooperation, and cargo delivery. FEBS J..

[20] Nachury M.V., Loktev A.V., Zhang Q., Westlake C.J., Peränen J., Merdes A., Slusarski D., Scheller R.H., Bazan J.F., Sheffield V. (2007). A Core Complex of BBS Proteins Cooperates with the GTPase Rab8 to Promote Ciliary Membrane Biogenesis. Cell.

[21] Lechtreck K.-F., Johnson E.C., Sakai T., Cochran D., Ballif B.A., Rush J., Pazour G., Ikebe M., Witman G.B. (2009). The Chlamydomonas reinhardtii BBSome is an IFT cargo required for export of specific signaling proteins from flagella. J. Cell Biol..

[22] Wingfield J., Lechtreck K.-F., Lorentzen E. (2018). Trafficking of ciliary membrane proteins by the intraflagellar transport/BBSome machinery. Essays Biochem..

[23] Benmerah A. (2013). The ciliary pocket. Curr. Opin. Cell Biol..

[24] Sorokin S.P. (1968). Reconstructions of Centriole Formation and Ciliogenesis in Mammalian Lungs. J. Cell Sci..

[25] Brown J.M., Witman G.B. (2014). Cilia and diseases. Bioscience.

[26] Pazour G., Dickert B.L., Vucica Y., Seeley E.S., Rosenbaum J.L., Witman G., Cole D.G. (2000). Chlamydomonas IFT88 and Its Mouse Homologue, Polycystic Kidney Disease Gene Tg737, Are Required for Assembly of Cilia and Flagella. J. Cell Biol..

[27] Anvarian Z., Mykytyn K., Mukhopadhyay S., Pedersen L., Christensen S.T. (2019). Cellular signalling by primary cilia in development, organ function and disease. Nat. Rev. Nephrol..

[28] Guemez-Gamboa A., Coufal N., Gleeson J.G. (2014). Primary Cilia in the Developing and Mature Brain. Neuron.

[29] May-Simera H., Nagel-Wolfrum K., Wolfrum U. (2017). Cilia—The sensory antennae in the eye. Prog. Retin. Eye Res..

[30] Pala R., A lOmari N., Nauli S.M. (2017). Primary Cilium-Dependent Signaling Mechanisms. Int. J. Mol. Sci..

[31] Falk N., Lösl M., Schröder N., Gießl A. (2015). Specialized Cilia in Mammalian Sensory Systems. Cells.

[32] Okamoto S., Chaya T., Omori Y., Kuwahara R., Kubo S., Sakaguchi H., Furukawa T. (2017). Ick ciliary kinase is essential for planar cell polarity formation in inner ear hair cells and hearing function. J. Neurosci..

[33] McGrath J., Somlo S., Makova S., Tian X., Brueckner M. (2003). Two Populations of Node Monocilia Initiate Left-Right Asymmetry in the Mouse. Cell.

[34] Nonaka S., Tanaka Y., Okada Y., Takeda S., Harada A., Kanai Y., Kido M., Hirokawa N. (1998). Randomization of Left–Right Asymmetry due to Loss of Nodal Cilia Generating Leftward Flow of Extraembryonic Fluid in Mice Lacking KIF3B Motor Protein. Cell.

[35] Sun S., Fisher R.L., Bowser S.S., Pentecost B.T., Sui H. (2019). Three-dimensional architecture of epithelial primary cilia. Proc. Natl. Acad. Sci. USA.

[36] Bernabé-Rubio M., Alonso M.A. (2017). Routes and machinery of primary cilium biogenesis. Cell. Mol. Life Sci..

[37] Gibbons I.R. (1963). Studies on the protein components of cilia from tetrahymena pyriformis. Proc. Natl. Acad. Sci. USA.

[38] Gibbons I.R., Rowe A.J. (1965). Dynein: A Protein with Adenosine Triphosphatase Activity from Cilia. Science.

[39] Toropova K., Zalyte R., Mukhopadhyay A., Mladenov M., Carter A.P., Roberts A.J. (2019). Structure of the dynein-2 complex and its assembly with intraflagellar transport trains. Nat. Struct. Mol. Biol..

[40] King S.M. (2016). Axonemal dynein arms. Cold Spring Harb. Persp. Biol..

[41] Roberts A.J., Kon T., Knight P.J., Sutoh K., Burgess S.A. (2013). Functions and mechanics of dynein motor proteins. Nat. Rev. Mol. Cell Biol..

[42] Reck-Peterson S.L., Redwine W.B., Vale R.D., Carter A.P. (2018). The cytoplasmic dynein transport machinery and its many cargoes. Nat. Rev. Mol. Cell Biol..

[43] Roberts A.J. (2018). Emerging mechanisms of dynein transport in the cytoplasm versus the cilium. Biochem. Soc. Trans..

[44] Grotjahn D.A., Lander G.C. (2019). Setting the dynein motor in motion: New insights from electron tomography. J. Biol. Chem..

[45] Pazour G.J., Wilkerson C.G., Witman G.B. (1998). A dynein light chain is essential for the retrograde particle movement of intraflagellar transport (ift). J. Cell Biol..

[46] Ocbina P.J., Eggenschwiler J.T., Moskowitz I., Anderson K.V. (2011). Complex interactions between genes controlling trafficking in primary cilia. Nat. Genet..

[47] Nachury M.V. (2014). How do cilia organize signalling cascades?. Philos. Trans. R Soc. Lond. B Biol. Sci..

[48] Jensen V.L., Lambacher N.J., Li C., Mohan S., Williams C.L., Inglis P.N., Yoder B.K., Blacque O.E., Leroux M.R. (2018). Role for intraflagellar transport in building a functional transition zone. EMBO Rep..

[49] Vuolo L., Stevenson N.L., Heesom K.J., Stephens D.J. (2018). Dynein-2 intermediate chains play crucial but distinct roles in primary cilia formation and function. eLife.

[50] Stepanek L., Pigino G. (2016). Microtubule doublets are double-track railways for intraflagellar transport trains. Science.

[51] Jordan M.A., Diener D.R., Stepanek L., Pigino G. (2018). The cryo-em structure of intraflagellar transport trains reveals how dynein is inactivated to ensure unidirectional anterograde movement in cilia. Nat. Cell Biol..

[52] Pazour G.J., Dickert B.L., Witman G.B. (1999). The dhc1b (dhc2) isoform of cytoplasmic dynein is required for flagellar assembly. J. Cell Biol..

[53] Hou Y., Witman G.B. (2015). Dynein and intraflagellar transport. Exp. Cell Res..

[54] Blisnick T., Buisson J., Absalon S., Marie A., Cayet N., Bastin P. (2014). The intraflagellar transport dynein complex of trypanosomes is made of a heterodimer of dynein heavy chains and of light and intermediate chains of distinct functions. Mol. Biol. Cell.

[55] Perrone C.A., Tritschler D., Taulman P., Bower R., Yoder B.K., Porter M.E. (2003). A novel dynein light intermediate chain colocalizes with the retrograde motor for intraflagellar transport at sites of axoneme assembly in chlamydomonas and mammalian cells. Mol. Biol. Cell.

[56] Grissom P.M., Vaisberg E.A., McIntosh J.R. (2002). Identification of a novel light intermediate chain (d2lic) for mammalian cytoplasmic dynein. Mol. Biol. Cell.

[57] Mikami A., Tynan S.H., Hama T., Luby-Phelps K., Saito T., Crandall J.E., Besharse J.C., Vallee R.B. (2002). Molecular structure of cytoplasmic dynein 2 and its distribution in neuronal and ciliated cells. J. Cell Sci..

[58] Huber C., Wu S., Kim A.S., Sigaudy S., Sarukhanov A., Serre V., Baujat G., Le Quan Sang K.H., Rimoin D.L., Cohn D.H. (2013). Wdr34 mutations that cause short-rib polydactyly syndrome type iii/severe asphyxiating thoracic dysplasia reveal a role for the nf-kappab pathway in cilia. Am. J. Hum. Genet..

[59] Gao D., Wang R., Li B., Yang Y., Zhai Z., Chen D.Y. (2009). Wdr34 is a novel tak1-associated suppressor of the il-1r/tlr3/tlr4-induced nf-kappab activation pathway. Cell Mol. Life Sci..

[60] McInerney-Leo A., Schmidts M., Cortes C., Leo P.J., Gener B., Courtney A.D., Gardiner B., Harris J.A., Lu Y., Marshall M. (2013). Short-Rib Polydactyly and Jeune Syndromes Are Caused by Mutations in WDR60. Am. J. Hum. Genet..

[61] Rompolas P., Pedersen L., Patel-King R.S., King S.M. (2007). Chlamydomonas FAP133 is a dynein intermediate chain associated with the retrograde intraflagellar transport motor. J. Cell Sci..

[62] Patel-King R.S., Gilberti R.M., Hom E.F.Y., King S.M. (2013). WD60/FAP163 is a dynein intermediate chain required for retrograde intraflagellar transport in cilia. Mol. Biol. Cell.

[63] Asante D., Stevenson N., Stephens D. (2014). Subunit composition of the human cytoplasmic dynein-2 complex. J. Cell Sci..

[64] Gholkar A., Senese S., Lo Y.-C., Capri J., Deardorff W.J., Dharmarajan H., Contreras E., Hodara E., Whitelegge J.P., Jackson P.K. (2015). Tctex1d2 associates with short-rib polydactyly syndrome proteins and is required for ciliogenesis. Cell Cycle.

[65] Norris D.P. (2012). Cilia, calcium and the basis of left-right asymmetry. BMC Biol..

[66] Spassky N., Meunier A. (2017). The development and functions of multiciliated epithelia. Nat. Rev. Mol. Cell Biol..

[67] Spassky N., Merkle F.T., Flames N., Tramontin A.D., Garcia-Verdugo J.M., Alvarez-Buylla A. (2005). Adult ependymal cells are postmitotic and are derived from radial glial cells during embryogenesis. J. Neurosci..

[68] Houtmeyers E., Gosselink R., Gayan-Ramirez G., Decramer M. (1999). Regulation of mucociliary clearance in health and disease. Eur. Respir. J..

[69] Afzelius B.A. (2004). Cilia-related diseases. J. Pathol..

[70] Hanasoge S., Hesketh P.J., Alexeev A. (2018). Metachronal motion of artificial magnetic cilia. Soft Matter.

[71] Bloodgood R.A. (2010). Sensory reception is an attribute of both primary cilia and motile cilia. J. Cell Sci..

[72] Lorenzo I.M., Liedtke W., Sanderson M.J., Valverde M.A. (2008). TRPV4 channel participates in receptor-operated calcium entry and ciliary beat frequency regulation in mouse airway epithelial cells. Proc. Natl. Acad. Sci. USA.

[73] Shah A.S., Ben-Shahar Y., Moninger T.O., Kline J.N., Welsh M.J. (2009). Motile Cilia of Human Airway Epithelia Are Chemosensory. Science.

[74] Teilmann S.C., Clement C.A., Thorup J., Byskov A.G., Christensen S.T. (2006). Expression and localization of the progesterone receptor in mouse and human reproductive organs. J. Endocrinol..

[75] Shao R., Weijdegard B., Fernandez-Rodriguez J., Egecioglu E., Zhu C., Andersson N., Thurin-Kjellberg A., Bergh C., Billig H. (2007). Ciliated epithelial-specific and regional-specific expression and regulation of the estrogen receptor-beta2 in the fallopian tubes of immature rats: A possible mechanism for estrogen-mediated transport process in vivo. Am. J. Physiol. Endocrinol. Metab..

[76] Shao R., Nutu M., Karlsson-Lindahl L., Benrick A., Weijdegard B., Lager S., Egecioglu E., Fernandez-Rodriguez J., Gemzell-Danielsson K., Ohlsson C. (2009). Downregulation of cilia-localized il-6r alpha by 17beta-estradiol in mouse and human fallopian tubes. Am. J. Physiol. Cell Physiol..

[77] Morimoto M., Liu Z., Cheng H.T., Winters N., Bader D., Kopan R. (2010). Canonical notch signaling in the developing lung is required for determination of arterial smooth muscle cells and selection of clara versus ciliated cell fate. J. Cell Sci..

[78] Meunier A., Azimzadeh J. (2016). Multiciliated cells in animals. Cold Spring Harb. Persp. Biol..

[79] Cibois M., Luxardi G., Chevalier B., Thomé V., Mercey O., Zaragosi L.-E., Barbry P., Pasini A., Marcet B., Kodjabachian L. (2015). BMP signalling controls the construction of vertebrate mucociliary epithelia. Development.

[80] Hiraoka K., Inada H., Yanai K., Osumi N. (2020). Bone Morphogenetic Proteins Inhibit Ciliogenesis of Ependymal Cells in Vitro. Tohoku J. Exp. Med..

[81] Ma L., Quigley I., Omran H., Kintner C. (2014). Multicilin drives centriole biogenesis via E2f proteins. Genes Dev..

[82] Zhao H., Zhu L., Zhu Y., Cao J., Li S., Huang Q., Xu T., Huang X., Yan X., Zhu X. (2013). The Cep63 paralogue Deup1 enables massive de novo centriole biogenesis for vertebrate multiciliogenesis. Nat. Cell Biol..

[83] Wallmeier J., Al-Mutairi D.A., Chen C.-T., Loges N.T., Pennekamp P., Menchen T., Ma L., Shamseldin H.E., Olbrich H., Dougherty G.W. (2014). Mutations in CCNO result in congenital mucociliary clearance disorder with reduced generation of multiple motile cilia. Nat. Genet..

[84] Purvis T.L., Hearn T., Spalluto C., Knorz V.J., Hanley K.P., Sanchez-Elsner T., Hanley N.A., Wilson D.I. (2010). Transcriptional regulation of the alstrom syndrome gene alms1 by members of the rfx family and sp1. Gene.

[85] Lemeille S., Paschaki M., Baas D., Morlé L., Duteyrat J.-L., Ait-Lounis A., Barras E., Soulavie F., Jerber J., Thomas J. (2020). Interplay of RFX transcription factors 1, 2 and 3 in motile ciliogenesis. Nucleic Acids Res..

[86] Lalioti M.-E., Arbi M., Loukas I., Kaplani K., Kalogeropoulou A., Lokka G., Kyrousi C., Mizi A., Georgomanolis T., Josipovic N. (2019). GemC1 governs multiciliogenesis through direct interaction and transcriptional regulation of p73. J. Cell Sci..

[87] Dehring D.A.K., Vladar E., Werner M.E., Mitchell J.W., Hwang P., Mitchell B. (2013). Deuterosome-Mediated Centriole Biogenesis. Dev. Cell.

[88] Al Jord A., Lemaître A.-I., Delgehyr N., Faucourt M., Spassky N., Meunier A. (2014). Centriole amplification by mother and daughter centrioles differs in multiciliated cells. Nat. Cell Biol..

[89] Mercey O., Levine M.S., Lo Mastro G.M., Rostaing P., Brotslaw E., Gomez V., Kumar A., Spassky N., Mitchell B.J., Meunier A. (2019). Massive centriole production can occur in the absence of deuterosomes in multiciliated cells. Nat. Cell Biol..

[90] Gomperts B.N., Gong-Cooper X., Hackett B.P. (2004). Foxj1 regulates basal body anchoring to the cytoskeleton of ciliated pulmonary epithelial cells. J. Cell Sci..

[91] Odate T., Takeda S., Narita K., Kawahara T.D. (2015). 9 + 0 and 9 + 2 cilia are randomly dispersed in the mouse node. Microscop.

[92] Loreng T.D., Smith E.F. (2016). The Central Apparatus of Cilia and Eukaryotic Flagella. Cold Spring Harb. Perspect. Biol..

[93] Zhao L., Hou Y., McNeill N.A., Witman G.B. (2019). The unity and diversity of the ciliary central apparatus. Philos. Trans. R. Soc. B Biol. Sci..

[94] Oda T., Yanagisawa H., Yagi T., Kikkawa M. (2014). Mechanosignaling between central apparatus and radial spokes controls axonemal dynein activity. J. Cell Biol..

[95] Gui M., Ma M., Sze-Tu E., Wang X., Koh F., Zhong E.D., Berger B., Davis J.H., Dutcher S.K., Zhang R. (2020). Structures of radial spokes and associated complexes important for ciliary motility. Nat. Struct. Mol. Biol..

[96] Osinka A., Poprzeczko M., Zielińska M., Fabczak H., Joachimiak E., Wloga D. (2019). Ciliary Proteins: Filling the Gaps. Recent Advances in Deciphering the Protein Composition of Motile Ciliary Complexes. Cells.

[97] Afzelius B. (1959). Electron Microscopy of the Sperm Tail Results Obtained with a New Fixative. J. Biophys. Biochem. Cytol..

[98] Wais-Steider J., Satir P. (1979). Effect of vanadate on gill cilia: Switching mechanism in ciliary beat. J. Supramol. Struct..

[99] Lin J., Nicastro D. (2018). Asymmetric distribution and spatial switching of dynein activity generates ciliary motility. Science.

[100] Kamiya R., Yagi T. (2014). Functional Diversity of Axonemal Dyneins as Assessed by in Vitro and in Vivo Motility Assays of Chlamydomonas Mutants. Zool. Sci..

[101] Pazour G., Agrin N.S., Walker B.L., Witman G.B. (2005). Identification of predicted human outer dynein arm genes: Candidates for primary ciliary dyskinesia genes. J. Med. Genet..

[102] Fabczak H., Osinka A. (2019). Role of the Novel Hsp90 Co-Chaperones in Dynein Arms’ Preassembly. Int. J. Mol. Sci..

[103] Dougherty G.W., Loges N.T., Klinkenbusch J.A., Olbrich H., Pennekamp P., Menchen T., Raidt J., Wallmeier J., Werner C., Westermann C. (2016). Dnah11 localization in the proximal region of respiratory cilia defines distinct outer dynein arm complexes. Am. J. Respir. Cell Mol. Biol..

[104] Fliegauf M., Olbrich H., Horvath J., Wildhaber J.H., Zariwala M.A., Kennedy M., Knowles M.R., Omran H. (2005). Mislocalization of DNAH5 and DNAH9 in Respiratory Cells from Patients with Primary Ciliary Dyskinesia. Am. J. Respir. Crit. Care Med..

[105] Viswanadha R., Sale W.S., Porter M.E. (2017). Ciliary Motility: Regulation of Axonemal Dynein Motors. Cold Spring Harb. Perspect. Biol..

[106] Höben I.M., Hjeij R., Olbrich H., Dougherty G.W., Nöthe-Menchen T., Aprea I., Frank D., Pennekamp P., Dworniczak B., Wallmeier J. (2018). Mutations in C11orf70 Cause Primary Ciliary Dyskinesia with Randomization of Left/Right Body Asymmetry Due to Defects of Outer and Inner Dynein Arms. Am. J. Hum. Genet..

[107] Pazour G.J., Quarmby L., Smith A.O., Desai P.B., Schmidts M. (2020). Cilia in cystic kidney and other diseases. Cell Sign..

[108] Chen X., Garcelon N., Neuraz A., Billot K., Lelarge M., Bonald T., Garcia H., Martin Y., Benoit V., Vincent M. (2019). Phenotypic similarity for rare disease: Ciliopathy diagnoses and subtyping. J. Biomed. Inf..

[109] Wheway G., Mitchison H.M., Genomics England Research C. (2019). Opportunities and challenges for molecular understanding of ciliopathies-the 100,000 genomes project. Front. Genet..

[110] Reiter J.F., Leroux M.R. (2017). Genes and molecular pathways underpinning ciliopathies. Nat. Rev. Mol. Cell Biol..

[111] Kulaga H.M., Leitch C.C., Eichers E.R., Badano J., Lesemann A., Hoskins B.E., Lupski J.R., Beales P.L., Reed R.R., Katsanis N. (2004). Loss of BBS proteins causes anosmia in humans and defects in olfactory cilia structure and function in the mouse. Nat. Genet..

[112] Tsang S.H., Aycinena A.R.P., Sharma T. (2018). Ciliopathy: Usher Syndrome. Atlas Inher. Ret. Deas..

[113] Pazour G.J. (2004). Intraflagellar transport and cilia-dependent renal disease: The ciliary hypothesis of polycystic kidney disease. J. Am. Soc. Nephrol..

[114] Yoder B.K., Hou X., Guay-Woodford L.M. (2002). The polycystic kidney disease proteins, polycystin-1, polycystin-2, polaris, and cystin, are co-localized in renal cilia. J. Am. Soc. Nephrol..

[115] Zhang M.-Z., Mai W., Li C., Cho S.-Y., Hao C., Moeckel G., Zhao R., Kim I., Wang J., Xiong H. (2004). PKHD1 protein encoded by the gene for autosomal recessive polycystic kidney disease associates with basal bodies and primary cilia in renal epithelial cells. Proc. Natl. Acad. Sci. USA.

[116] Otto E.A., Schermer B., Obara T., O’Toole J.F., Hiller K.S., Mueller A.M., Ruf R.G., Hoefele J., Beekmann F., Landau D. (2003). Mutations in invs encoding inversin cause nephronophthisis type 2, linking renal cystic disease to the function of primary cilia and left-right axis determination. Nat. Genet..

[117] Sheffield V.C., Carml R., Kwltek-Black A., Rokhlina T., Nishlmura D., Duyk G.M., Elbedour K., Sunden S.L., Stone E.M. (1994). Identification of a Bardet-Biedl syndrome locus on chromosome 3 and evaluation of an efficient approach to homozygosity mapping. Hum. Mol. Genet..

[118] Waters A.M., Beales P.L. (2011). Ciliopathies: An expanding disease spectrum. Pediatr. Nephrol..

[119] Antony D., Nampoory N., Bacchelli C., Melhem M., Wu K., James C., Beales P.L., Hubank M., Thomas D., Mashankar A. (2017). Exome sequencing for the differential diagnosis of ciliary chondrodysplasias: Example of a WDR35 mutation case and review of the literature. Eur. J. Med. Genet..

[120] Patel H., Li J., Herrero A., Kroboth J., Byron A., Von Kriegsheim A., Brunton V., Carragher N., Hurd T., Frame M. (2020). Novel roles of PRK1 and PRK2 in cilia and cancer biology. Sci. Rep..

[121] Knowles M.R., Zariwala M., Leigh M. (2016). Primary Ciliary Dyskinesia. Clin. Chest Med..

[122] Wallmeier J., Frank D., Shoemark A., Nothe-Menchen T., Cindric S., Olbrich H., Loges N.T., Aprea I., Dougherty G.W., Pennekamp P. (2019). De novo mutations in foxj1 result in a motile ciliopathy with hydrocephalus and randomization of left/right body asymmetry. Am. J. Hum. Genet..

[123] Powles-Glover N. (2014). Cilia and ciliopathies: Classic examples linking phenotype and genotype—An overview. Reprod. Toxicol..

[124] Oud M.M., Lamers I.J.C., Arts H.H. (2016). Ciliopathies: Genetics in Pediatric Medicine. J. Pediatr. Genet..

[125] Olson A.J., Krentz A.D., Finta K.M., Okorie U.C., Haws R.M. (2019). Thoraco-Abdominal Abnormalities in Bardet-Biedl Syndrome: Situs Inversus and Heterotaxy. J. Pediatr..

[126] Meng C., Zhang K.H., Ma J., Gao X., Yu K., Zhang H.Y., Wang Y., Zhang Z.X., Li W.G., Liu Y. (2017). Clinical and genetic analysis of a family with Joubert syndrome type 10 caused by OFD1 gene mutation. Zhonghua er ke za zhi Chin. J. Pediatr..

[127] Porter M.E., Bower R., Knott J.A., Byrd P., Dentler W. (1999). Cytoplasmic dynein heavy chain 1b is required for flagellar assembly in chlamydomonas. Mol. Biol. Cell.

[128] Wu C., Li J., Peterson A., Tao K., Wang B. (2017). Loss of dynein-2 intermediate chain Wdr34 results in defects in retrograde ciliary protein trafficking and Hedgehog signaling in the mouse. Hum. Mol. Genet..

[129] Baujat G., Huber C., El Hokayem J., Caumes R., Thanh C.D.N., David A., Delezoide A.-L., Dieux-Coeslier A., Estournet B., Francannet C. (2013). Asphyxiating thoracic dysplasia: Clinical and molecular review of 39 families. J. Med. Genet..

[130] Dagoneau N., Goulet M., Genevieve D., Sznajer Y., Martinovic J., Smithson S., Huber C., Baujat G., Flori E., Tecco L. (2009). Dync2h1 mutations cause asphyxiating thoracic dystrophy and short rib-polydactyly syn-drome, type iii. Am. J. Hum. Genet..

[131] Schmidts M., Vodopiutz J., Christou-Savina S., Cortes C., McInerney-Leo A.M., Emes R., Arts H.H., Tüysüz B., D’Silva J., Leo P.J. (2013). Mutations in the Gene Encoding IFT Dynein Complex Component WDR34 Cause Jeune Asphyxiating Thoracic Dystrophy. Am. J. Hum. Genet..

[132] Taylor S.P., Dantas T.J., Duran I., Wu S., Lachman R.S., Nelson S.F., Cohn D.H., Vallee R.B., Krakow D., University of Washington Center for Mendelian Genomics Consortium (2015). Mutations in DYNC2LI1 disrupt cilia function and cause short rib polydactyly syndrome. Nat. Commun..

[133] Schmidts M., Hou Y., Cortes C.R., Mans D.A., Huber C., Boldt K., Patel M., van Reeuwijk J., Plaza J.M., van Beersum S.E. (2015). Tctex1d2 mutations underlie jeune asphyxiating thoracic dystrophy with impaired retrograde intraflagellar transport. Nat. Commun..

[134] Schmidts M. (2015). Clinical genetics and pathobiology of ciliary chondrodysplasias. J. Pediatr. Genet..

[135] Schmidts M., Arts H.H., Bongers E.M., Yap Z., Oud M.M., Antony D., Duijkers L., Emes R.D., Stalker J., Yntema J.B. (2013). Exome sequencing identifies dync2h1 mutations as a common cause of asphyxiating thoracic dystrophy (jeune syndrome) without major polydactyly, renal or retinal involvement. J. Med. Genet..

[136] Halbritter J., Bizet A., Schmidts M., Porath J.D., Braun D.A., Gee H.Y., McInerney-Leo A., Krug P., Filhol E., Davis E. (2013). Defects in the IFT-B Component IFT172 Cause Jeune and Mainzer-Saldino Syndromes in Humans. Am. J. Hum. Genet..

[137] Vig A., Poulter J.A., Ottaviani D., Tavares E., Toropova K., Tracewska A.M., Mollica A., Kang J., Kehelwathugoda O., Genomics England Research Consortium (2020). DYNC2H1 hypomorphic or retina-predominant variants cause nonsyndromic retinal degeneration. Genet. Med..

[138] Thiel C., Kessler K., Gießl A., Dimmler A., Shalev S.A., von der Haar S., Zenker M., Zahnleiter D., Stöss H., Beinder E. (2011). NEK1 Mutations Cause Short-Rib Polydactyly Syndrome Type Majewski. Am. J. Hum. Genet..

[139] Mill P., Lockhart P.J., Fitzpatrick E., Mountford H., Hall E., Reijns M., Keighren M., Bahlo M., Bromhead C.J., Budd P. (2011). Human and Mouse Mutations in WDR35 Cause Short-Rib Polydactyly Syndromes Due to Abnormal Ciliogenesis. Am. J. Hum. Genet..

[140] Niceta M., Margiotti K., Digilio M., Guida V., Bruselles A., Pizzi S., Ferraris A., Memo L., Laforgia N., Dentici M. (2017). Biallelic mutations in DYNC2LI1 are a rare cause of Ellis-van Creveld syndrome. Clin. Genet..

[141] Solaguren-Beascoa M., Bujakowska K.M., Mejecase C., Emmenegger L., Orhan E., Neuille M., Mohand-Said S., Condroyer C., Lancelot M.E., Michiels C. (2020). Wdr34, a candidate gene for non-syndromic rod-cone dystrophy. Clin. Genet..

[142] Schmidts M., Frank V., Eisenberger T., Al Turki S., Bizet A.A., Antony D., Rix S., Decker C., Bachmann N., Bald M. (2013). Combined ngs approaches identify mutations in the intraflagellar transport gene ift140 in skeletal ciliopathies with early progressive kidney disease. Hum. Mut..

[143] Echelard Y., Epstein D.J., St-Jacques B., Shen L., Mohler J., McMahon J.A., McMahon A.P. (1993). Sonic hedgehog, a member of a family of putative signaling molecules, is implicated in the regulation of CNS polarity. Cell.

[144] Bitgood M.J., Shen L., McMahon A.P. (1996). Sertoli cell signaling by Desert hedgehog regulates the male germline. Curr. Biol..

[145] Wijgerde M., Ooms M., Hoogerbrugge J.W., Grootegoed J.A. (2005). Hedgehog Signaling in Mouse Ovary: Indian Hedgehog and Desert Hedgehog from Granulosa Cells Induce Target Gene Expression in Developing Theca Cells. Endocrinology.

[146] Chiang C., Litingtung Y., Lee E., Young K.E., Corden J.L., Westphal H., Beachy P.A. (1996). Cyclopia and defective axial patterning in mice lacking Sonic hedgehog gene function. Nat. Cell Biol..

[147] St-Jacques B., Hammerschmidt M., McMahon A.P. (1999). Indian hedgehog signaling regulates proliferation and differentiation of chondrocytes and is essential for bone formation. Genes Dev..

[148] Tempe D., Casas M., Karaz S., Blanchet-Tournier M.F., Concordet J.P. (2006). Multisite protein kinase a and glycogen synthase kinase 3beta phosphorylation leads to gli3 ubiquitination by scfbetatrcp. Mol. Cell Biol..

[149] Carney T.J., Ingham P.W. (2013). Drugging Hedgehog: Signaling the pathway to translation. BMC Biol..

[150] Onoufriadis A., Paff T., Antony D., Shoemark A., Micha D., Kuyt B., Schmidts M., Petridi S., Dankert-Roelse J.E., Haarman E.G. (2013). Splice-Site Mutations in the Axonemal Outer Dynein Arm Docking Complex Gene CCDC114 Cause Primary Ciliary Dyskinesia. Am. J. Hum. Genet..

[151] Fliegauf M., Benzing T., Omran H. (2007). When cilia go bad: Cilia defects and ciliopathies. Nat. Rev. Mol. Cell Biol..

[152] Noone P.G., Leigh M.W., Sannuti A., Minnix S.L., Carson J.L., Hazucha M., Zariwala M.A., Knowles M.R. (2004). Primary ciliary dyskinesia: Diagnostic and phenotypic features. Am. J. Respir. Crit. Care Med..

[153] Best S., Shoemark A., Rubbo B., Patel M.P., Fassad M., Dixon M., Rogers A.V., Hirst R.A., Rutman A., Ollosson S. (2018). Risk factors for situs defects and congenital heart disease in primary ciliary dyskinesia. Thorax.

[154] Kennedy M.P., Omran H., Leigh M.W., Dell S., Morgan L., Molina P.L., Robinson B.V., Minnix S.L., Olbrich H., Severin T. (2007). Congenital Heart Disease and Other Heterotaxic Defects in a Large Cohort of Patients With Primary Ciliary Dyskinesia. Circulation.

[155] Shapiro A.J., Davis S.D., Ferkol T., Dell S.D., Rosenfeld M., Olivier K.N., Sagel S.D., Milla C., Zariwala M.A., Wolf W. (2014). Laterality defects other than situs inversus totalis in primary ciliary dyskinesia: Insights into situs ambiguus and heterotaxy. Chest.

[156] Deng H., Xia H., Deng S. (2014). Genetic basis of human left–right asymmetry disorders. Expert Rev. Mol. Med..

[157] Zuckerman H.S., Wurtzebach L.R. (1951). Kartagener’s triad, review of the literature and report of a case. Dis. Chest.

[158] Loges N.T., Antony D., Maver A., Deardorff M.A., Gulec E.Y., Gezdirici A., Nothe-Menchen T., Hoben I.M., Jelten L., Frank D. (2018). Recessive dnah9 loss-of-function mutations cause laterality defects and subtle respiratory ciliary-beating defects. Am. J. Hum. Genet..

[159] Mitchison H.M., Schmidts M., Loges N.T., Freshour J., Dritsoula A., Hirst R.A., O’Callaghan C., Blau H., Al Dabbagh M., Olbrich H. (2012). Mutations in axonemal dynein assembly factor DNAAF3 cause primary ciliary dyskinesia. Nat. Genet..

[160] Olbrich H., Schmidts M., Werner C., Onoufriadis A., Loges N.T., Raidt J., Banki N.F., Shoemark A., Burgoyne T., Al Turki S. (2012). Recessive HYDIN Mutations Cause Primary Ciliary Dyskinesia without Randomization of Left-Right Body Asymmetry. Am. J. Hum. Genet..

[161] Antony D., Becker-Heck A., Zariwala M.A., Schmidts M., Onoufriadis A., Forouhan M., Wilson R., Taylor-Cox T., Dewar A., Jackson C. (2013). Mutations in CCDC39 and CCDC 40 are the Major Cause of Primary Ciliary Dyskinesia with Axonemal Disorganization and Absent Inner Dynein Arms. Hum. Mutat..

[162] Gibbons B.H., Gibbons I.R. (1973). The Effect of Partial Extraction of Dynein Arms on the Movement of Reactivated Sea-Urchin Sperm. J. Cell Sci..

[163] Kamiya R., Okamoto M. (1985). A mutant of *Chlamydomonas reinhardtii* that lacks the flagellar outer dynein arm but can swim. J. Cell Sci..

[164] Brokaw C.J., Kamiya R. (1987). Bending patterns of *Chlamydomonas flagella*: IV. Mutants with defects in inner and outer dynein arms indicate differences in dynein arm function. Cell Motil. Cytoskelet..

[165] Wilkerson C.G., King S.M., Koutoulis A., Pazour G., Witman G. (1995). The 78,000 M(r) intermediate chain of Chlamydomonas outer arm dynein isa WD-repeat protein required for arm assembly. J. Cell Biol..

[166] Pennarun G., Escudier E., Chapelin C., Bridoux A.-M., Cacheux V., Roger G., Clément A., Goossens M., Amselem S., Duriez B. (1999). Loss-of-Function Mutations in a Human Gene Related to Chlamydomonas reinhardtii Dynein IC78 Result in Primary Ciliary Dyskinesia. Am. J. Hum. Genet..

[167] Omran H., Häffner K., Völkel A., Kuehr J., Ketelsen U.-P., Ross U.-H., Konietzko N., Wienker T., Brandis M., Hildebrandt F. (2000). Homozygosity Mapping of a Gene Locus for Primary Ciliary Dyskinesia on Chromosome 5p and Identification of the Heavy Dynein ChainDNAH5as a Candidate Gene. Am. J. Respir. Cell Mol. Biol..

[168] Poprzeczko M., Bicka M., Farahat H., Bazan R., Osinka A., Fabczak H., Joachimiak E., Wloga D. (2019). Rare human diseases: Model organisms in deciphering the molecular basis of primary ciliary dyskinesia. Cells.

[169] Leigh M.W., Horani A., Kinghorn B., O’Connor M.G., Zariwala M.A., Knowles M.R. (2019). Primary ciliary dyskinesia (PCD): A genetic disorder of motile cilia. Transl. Sci. Rare Dis..

[170] Rossman C.M., Forrest J.B., Lee R.M., Newhouse M.T. (1980). The dyskinetic cilia syndrome. Ciliary motility in immotile cilia syndrome. Chest.

[171] Olbrich H., Haffner K., Kispert A., Volkel A., Volz A., Sasmaz G., Reinhardt R., Hennig S., Lehrach H., Konietzko N. (2002). Mutations in dnah5 cause primary ciliary dyskinesia and randomization of left-right asymmetry. Nat. Genet..

[172] Duriez B., Duquesnoy P., Escudier E., Bridoux A.-M., Escalier D., Rayet I., Marcos E., Vojtek A.-M., Bercher J.-F., Amselem S. (2007). A common variant in combination with a nonsense mutation in a member of the thioredoxin family causes primary ciliary dyskinesia. Proc. Natl. Acad. Sci. USA.

[173] Bustamante-Marin X.M., Horani A., Stoyanova M., Charng W.-L., Bottier M., Sears P.R., Yin W.-N., Daniels L.A., Bowen H., Conrad D.F. (2020). Mutation of CFAP57, a protein required for the asymmetric targeting of a subset of inner dynein arms in Chlamydomonas, causes primary ciliary dyskinesia. PLoS Genet..

[174] O’Callaghan C., Rutman A., Williams G.M., Hirst R.A. (2011). Inner dynein arm defects causing primary ciliary dyskinesia: Repeat testing required. Eur. Respir. J..

[175] Merveille A.C., Davis E.E., Becker-Heck A., Legendre M., Amirav I., Bataille G., Belmont J., Beydon N., Billen F., Clement A. (2011). Ccdc39 is required for assembly of inner dynein arms and the dynein regulatory com-plex and for normal ciliary motility in humans and dogs. Nat. Genet..

[176] Becker-Heck A., Zohn I.E., Okabe N., Pollock A., Lenhart K.B., Sullivan-Brown J., McSheene J., Loges N.T., Olbrich H., Haeffner K. (2010). The coiled-coil domain containing protein CCDC40 is essential for motile cilia function and left-right axis formation. Nat. Genet..

[177] Ibañez-Tallon I., Pagenstecher A., Fliegauf M., Olbrich H., Kispert A., Ketelsen U.-P., North A., Heintz N., Omran H. (2004). Dysfunction of axonemal dynein heavy chain Mdnah5 inhibits ependymal flow and reveals a novel mechanism for hydrocephalus formation. Hum. Mol. Genet..

[178] Lucas J., Adam E.C., Goggin P.M., Jackson C., Powles-Glover N., Patel S.H., Humphreys J., Fray M.D., Falconnet E., Blouin J.-L. (2011). Static respiratory cilia associated with mutations in Dnahc11/DNAH11: A mouse model of PCD. Hum. Mut..

[179] Tan S., Rosenthal J., Zhao X.-Q., Francis R., Chatterjee B., Sabol S.L., Linask K.L., Bracero L., Connelly P.S., Daniels M.P. (2007). Heterotaxy and complex structural heart defects in a mutant mouse model of primary ciliary dyskinesia. J. Clin. Investig..

[180] Bartoloni L., Blouin J.-L., Pan Y., Gehrig C., Maiti A.K., Scamuffa N., Rossier C., Jorissen M., Armengot M., Meeks M. (2002). Mutations in the DNAH11 (axonemal heavy chain dynein type 11) gene cause one form of situs inversus totalis and most likely primary ciliary dyskinesia. Proc. Natl. Acad. Sci. USA.

[181] Lee L., Ostrowski L.E. (2020). Motile cilia genetics and cell biology: Big results from little mice. Cell. Mol. Life Sci..

[182] Coles J.L., Thompson J., Horton K.L., Hirst R.A., Griffin P., Williams G.M., Goggin P., Doherty R., Lackie P.M., Harris A. (2020). A Revised Protocol for Culture of Airway Epithelial Cells as a Diagnostic Tool for Primary Ciliary Dyskinesia. J. Clin. Med..

[183] Collins S.A., Gove K., Walker W., Lucas J.S. (2014). Nasal nitric oxide screening for primary ciliary dyskinesia: Systematic review and meta-analysis. Eur. Respir. J..

[184] Dalrymple R.A., Kenia P. (2018). European Respiratory Society guidelines for the diagnosis of primary ciliary dyskinesia: A guideline review. Arch. Dis. Child. Educ. Pr. Ed..

[185] Boone M., Smits A., Cuppens H., Jaspers M., Proesmans M., Dupont L.J., Vermeulen F.L., Van Daele S., Malfroot A., Godding V. (2014). Primary ciliary dyskinesia: Critical evaluation of clinical symptoms and diagnosis in patients with normal and abnormal ultrastructure. Orphanet J. Rare Dis..

[186] Shoemark A., Frost E., Dixon M., Ollosson S., Kilpin K., Patel M., Scully J., Rogers A.V., Mitchison H.M., Bush A. (2017). Accuracy of Immunofluorescence in the Diagnosis of Primary Ciliary Dyskinesia. Am. J. Respir. Crit. Care Med..

